# The Gibbs Paradox: Early History and Solutions

**DOI:** 10.3390/e20060443

**Published:** 2018-06-06

**Authors:** Olivier Darrigol

**Affiliations:** UMR SPHere, CNRS, Université Denis Diderot, 75013 Paris, France; darrigol@univ-paris-diderot.fr

**Keywords:** Gibbs paradox, mixing, entropy, irreversibility, thermochemistry

## Abstract

This article is a detailed history of the Gibbs paradox, with philosophical morals. It purports to explain the origins of the paradox, to describe and criticize solutions of the paradox from the early times to the present, to use the history of statistical mechanics as a reservoir of ideas for clarifying foundations and removing prejudices, and to relate the paradox to broad misunderstandings of the nature of physical theory.

## 1. Introduction

The history of thermodynamics has three famous “paradoxes”: Josiah Willard Gibbs’s mixing paradox of 1876, Josef Loschmidt reversibility paradox of the same year, and Ernst Zermelo’s recurrence paradox of 1896. The second and third revealed contradictions between the law of entropy increase and the properties of the underlying molecular dynamics. They prompted Ludwig Boltzmann to deepen the statistical understanding of thermodynamic irreversibility. The Gibbs paradox—first called a paradox by Pierre Duhem in 1892—denounced a violation of the continuity principle: the mixing entropy of two gases (to be defined in a moment) has the same finite value no matter how small the difference between the two gases, even though common sense requires the mixing entropy to vanish for identical gases (you do not really mix two identical substances). Although this paradox originally belonged to purely macroscopic thermodynamics, Gibbs perceived kinetic-molecular implications and James Clerk Maxwell promptly followed him in this direction. From Gibbs to the present, the Gibbs paradox has tested the foundations of thermodynamics, both at the macroscopic and at the kinetic-molecular level.

A few lessons will be drawn from this history. First, the Gibbs paradox needs disambiguation. Different paradoxes have to be distinguished according to the theoretical level at which they occur. Second, most of the solutions that were proposed in the abundant literature on the paradox since the advent of quantum mechanics have (mostly unknown) antecedents in the nineteenth century. Third and most important, the paradox (in its various forms) cannot be solved without delving deeper into the foundations of thermodynamics and statistical mechanics than is usually done. Before any sound discussion, three foundational issues need to be addressed: the relevance of stationary ensembles to describe the equilibrium of a single system, the physical meaning of statistico-mechanical probabilities, and their relation to entropy. Here too, history helps because the founding fathers Boltzmann and Albert Einstein explained these points in a much fuller manner than his usually done in modern texts on statistical thermodynamics. Lastly (see also [1]), the history of the paradox leads us to reject common prejudices about the extensivity of entropy (even for gases), about the impossibility of solving the paradox in a purely classical context, and about the role of quantum indistinguishability in solving the paradox.

The first purpose of this essay is to retrace the early history of the Gibbs paradox, including the thermodynamic and chemical contexts in which its chief ingredients emerged, early formulations and attempted solutions from Gibbs to Max Planck, and parallel developments that implicitly bear on the paradox, for instance Boltzmann’s study of chemical equilibrium and Paul Ehrenfest’s rejection of extensive entropies. These two physicists certainly knew about the Gibbs paradox (Boltzmann used the last sentence of Gibbs’s discussion, “The impossibility of an uncompensated decrease of entropy seems to be reduced to improbability”, as an epigraph to the second volume of his lectures on gas theory). Yet they did not discuss it in their writings, possibly because they knew the paradox disappeared in their own concept of mixing.

Section 2 of this essay sets the stage for Gibbs’s remarkable theory of chemical equilibrium, in which he enunciated his eponymous paradox in April 1876. In the years preceding this theory, thermodynamics was still a developing theory whose principles and concepts were often misunderstood. Violations of the second law were still contemplated; and entropy, which Rudolf Clausius introduced in 1865, was commonly regarded as a mysterious and unnecessary concept. The first discussions of work produced by mixing (Loschmidt in 1869, Rayleigh in 1875) and the first applications of thermodynamics to chemistry (Loschmidt in 1869, Horstmann in 1873) occurred in this unstable conceptual environment. They involved the basic idea that chemical equilibrium depends not only on the energy (or enthalpy) balance of the reaction but also on the entropy balance, which itself depends on the mixing entropy in the case of reactions involving gases or solutes.

Section 3 is devoted to Gibbs’s memoir “On the equilibrium of heterogeneous substances” (1875–1878) and the included paradox. Special attention is given to the two basic presuppositions of the paradox: Gibbs’s rule for computing the entropy of a gas mixture, and entropy extensivity. Gibbs’s discussion of the paradox has several facets, including the idea that entropy depends on sensible properties only and the suggestion that the entropy law has statistical value only. 

Section 4 recounts how various authors discussed the interdiffusion of gases with respect to the second law in the three following years, thus preparing further discussion of the Gibbs paradox. James Clerk Maxwell used the Gibbs paradox to reveal the subjective side of entropy determinations; Simon Tolver Preston claimed he could violate the second law by diffusion through porous membranes; and Ludwig Boltzmann refuted Preston’s claim in a thorough analysis of the preconditions for computing the mixing entropy. He thereby introduced the concept of semipermeable wall, which soon became standard in this domain. 

Section 5 is about the first explicit discussions of the Gibbs paradox after Gibbs and Maxwell, under the pen of Carl Neumann, Pierre Duhem, and Otto Wiedeburg in the 1890s. This last author is often credited with the phrase “Gibbs paradox,” for it occurs in the very title of his article. In reality, Wiedeburg borrowed the name from Duhem, who himself benefitted from the outstanding clarity of Neumann’s relevant analysis. Wiedeburg’s chief innovation was the idea, soon publicized by Max Planck, that the paradox implies the essentially discrete character of chemical differences. 

Section 6 is about entropy extensivity in statistico-mechanical context, with the (in)famous N! division that several authors introduced in thermodynamic probabilities in order that the associated entropy be extensive. There were two conflicting conceptions. In the first, inaugurated by Boltzmann in 1883 and extended by Ehrenfest in 1920 in quantum context, the N! division is justified through the expression of the combinatorial probability for molecules whose number varies through the exchange of distinguishable atoms in a chemical reaction. In the second conception, introduced by Gibbs in 1902 and extended by Hugo Tetrode (1912) and Max Planck (1916) in quantum context, the N! division is regarded as a natural consequence of the perfect identity or “indistinguishability” of the molecules in the holistic, ensemble-based approach to thermodynamic equilibrium. Ehrenfest (and Albert Einstein) denied that Gibbs and Planck had a proper justification for the N! division. Einstein’s new gas theory of 1924 changed the game by spontaneously yielding extensive entropies and thus aggravating the Gibbs paradox. In 1832, Johann von Neumann nonetheless claimed to have solved the paradox in quantum-mechanical context.

Section 7 is a synthetic discussion of the Gibbs paradox based on its early history. Three different paradoxes are distinguished. The first belongs to a proto-thermodynamics not yet equipped with the entropy concept; it concerns the maximal work that can be obtained by the interdiffusion of two gases; and it is solved by noting that this work depends on the contingent existence of a separation process. The second paradox belongs to macroscopic thermodynamics including the entropy concept. It may be solved in four different manners: by making entropy and the state parameters of which it is a function depend on the contingent existence of a separation process (Gibbs, Maxwell, Jaynes), by making entropy depend on a universe of ideal operations (Bridgman), by invoking an intrinsic discontinuity in chemical differences (Wiedeburg, Planck), by allowing non-extensive entropies. The third paradox belongs to statistical thermodynamics. In classical context, it can be solved by giving the mixing entropy the same value for different and for identical gases (early Einstein, Grad, Dieks) or by broadly denying that the theory implies entropy extensivity (Ehrenfest, van Kampen, Jaynes). In quantum context, it can still be solved through the latter means (van Kampen), or we may rely on quantum-mechanical means. In this second option, one may argue that quantum indistinguishability introduces an essential discontinuity between the case of slightly different gases and the case of identical gases (Schrödinger), or, better, one may show that quantum theory provides just the right amount of continuity and discontinuity between the two cases (von Neumann and Landé, whose considerations are here improved).

The multiplicity of solutions proposed for the two last versions of the paradox is the sign that there are different ways of conceiving the foundations of thermodynamics and statistical mechanics. In particular, various authors disagree on whether statistical mechanics can determine the way in which the entropy of a confined gas depends on the number of molecules. In Section 7, it is argued that the latter determination becomes possible if statistical mechanics is complemented with a fluctuation principle introduced by Einstein. With this principle it becomes possible to exploit the fluctuations of a given system around equilibrium to derive the equilibrium entropy of a partial system.

Section 8, the last of this essay, is an attempt to relate the Gibbs paradox to broad misunderstandings of the way in which physical theory connects to the experimental world. The essential idea is that the symbolic universe of a theory acquires physical content only through an evolving class of interpretive schemes that provide blue prints for conceivable experiments. The systems and processes of the symbolic universe are free-floating mathematical creations, and naive projection of some of their properties onto the interpretive level leads to paradoxes.

**Figure 1:**
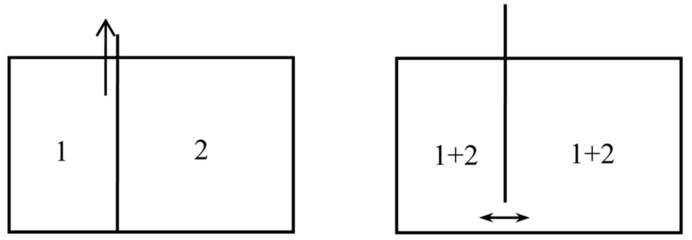
The spontaneous, natural mixing of two gases. The gases 1 and 2, originally in two separate compartments, are allowed to diffuse into each other by lifting the wall that separates them.

In the following, the now standard notation for thermodynamic and statistico-mechanical quantities are used. In particular, the Boltzmann constant $k_{B}$ is inserted in the statistical entropy formulas, contrary to Boltzmann’s practice (he measured temperatures in energy units). A process is called *reversible* when it is both reversible in the ordinary sense and quasistatic. *Natural mixing* is an isothermal mixing process in which the volume of the mixture at the end of the process is equal to the sum of the volumes of the two gases before mixture. The entropy created during such a process is called the *entropy of mixing* or *mixing entropy*. Natural mixing may occur spontaneously and irreversibly, when a partition between two chambers is removed (see Figure 1). Or it may be done reversibly, in which case it produces work (see below, Section 2.4). Reversible, isothermal mixing may be non-natural; for instance, when the volumes of the two unmixed gases are equal, it may be done in such a manner that this volume is equal to the volume of the mixture (see below, Section 4.4, Figure 10). In the latter case and for non-interacting gases, by Gibbs’s *mixing rule* the entropy of the mixture is equal to the sum of the entropies of the unmixed gases (the mixing is isentropic).

## 2. Diffusion and Dissipation before Gibbs

### 2.1. Some Background: Clausius’s Axiom, the Second Law, Entropy, Disgregation

Modern thermodynamics emerged around 1850 from James Joule’s empirical proof of the quantitative equivalence between heat and work and from Sadi Carnot’s general theorem, according to which the maximum efficiency of a cyclic engine producing work while borrowing heat from a hot source and returning it to a cold source is a universal function of the temperatures of the two sources (see [2]). Carnot based his derivation of the theorem on the impossibility of perpetual motion and on the conservation of heat (“caloric fluid”) during its “fall” from the hot source to the cold source, in evident analogy with hydraulic machines. In 1850, Rudolf Clausius [3] solved the contradiction between the latter assumption and Joule’s equivalence between heat and work by assuming that only part of the heat from the hot source was transferred to the cold source, the rest being converted into heat. In the derivation of Carnot’s theorem, Clausius replaced the impossibility of perpetual motion with the impossibility of the transfer of heat from a cold source to a warm source without compensation in the environment. William Thomson [4] soon offered an alternative derivation based on the impossibility of producing work from a single source of heat.

In early thermodynamics (as Thomson called the new science), the “first law” was the equivalence between heat and work or, in Thomson’s sharper formulation, the proportionality of the heat and work exchanged by a system with its environment during any cycle of operations on this system. The “second law” was most commonly the name given to Carnot’s theorem, justified by “Clausius’s axiom” regarding the irreversibility of spontaneous heat transfer or “Thomson’s axiom” regarding the exclusion of monothermal engines (By definition, a monothermal engine produces work in a cycle of operations during which it exchanges heat with bodies all at the same temperature). There was some uncertainty regarding the precise meaning of these axioms. In particular, it was not clear what could serve as a compensation for the transfer of heat from a cold to a warm body or what could serve as a compensation for the production of work from a single source of heat. Was it just work in the former case? Was it just the absorption of heat by a colder source in the latter case?

These obscurities affected the reception of the theory without hampering progress in the hand of its creators. Thomson was soon able to apply thermodynamics well beyond the original context of heat engines, to thermoelectric, thermoelastic, and Galvanic phenomena. Yet no one in those early years tried to apply the theory to chemical processes, presumably because chemical reactions generally involved irreversible changes that seemed to elude quantitative applications of the laws of thermodynamics. These applications were usually done through imaginary reversible cyclic processes involving the phenomena of interest, or through the identity

(1) $$\oint \frac{\delta Q}{T}=0,$$

which Thomson [4] and Clausius [5] independently derived in 1854 for a reversible cycle of operations on a system exchanging the heat δQ with sources at the absolute temperature *T* defined through Carnot’s theorem.

For a better intuition of the principles of thermodynamics, Clausius relied on Carnot’s original analogy between the fall of caloric and the fall of water in a hydraulic machine. Accordingly, the total work δA obtainable from a substance at temperature *T* should be proportional to *T* (just as the work obtainable from a water reservoir depends on the height of this reservoir). In 1862, Clausius [6] expressed this condition as

(2) δA≡dw−δW=TdY,

wherein dw is the work done by the internal forces of the substance (the increase of its potential energy), −δW the work done on its environment, and *Y* what Clausius called the *disgregation*, for it gave “the degree in which the molecules of the body are dispersed” (see [7,8]). The disgregation has not survived modern thermodynamics, in part because it involves the molecular work dw, which is not a purely macroscopic notion, and mostly because the more convenient entropy concept superseded it. However, in the 1860s and 1870s disgregation and entropy were still in competition for the few consumers of abstract concepts or hidden entities in thermodynamics.

Clausius [9] introduced the *entropy* of a system in 1865 as the integral of the differential δQ/T over any reversible transformation of the system from a fixed reference state to the state under consideration. He then proved that during an irreversible transformation the sum of the entropy of the system and the entropies of the sources with which it exchanges heat is always increasing. He concluded his memoir with the two statements:

(1) The energy of the world is constant.
(2) The entropy of the world tends to a maximum.

For a better intuition of entropy, Clausius related it to his earlier concept of disgregation. Introducing the “free heat” $\bar{K}$ which is the average kinetic energy of the molecular system, the variation of the total energy *U* of the system during an infinitesimal transformation can be written as

(3) $$dU=\delta W+\delta Q=dw+d\bar{K}.$$

Together with Equation (2), this implies

(4) $$dS=dY+d\bar{K}/T.$$

The entropy thus appears to be the sum of the disgregation of the system and of a function of the temperature only.

Despite the formal appeal of a quantity that naturally occurs as the integral of the differential δQ/T and despite the attractive generality of the entropy law enunciated by Clausius in parallel with the energy law, it took a long time for entropy to become a main-stream concept of thermodynamics. British physicists preferred Thomson’s more intuitive notions of dissipated energy and available work, to which we will return in a moment. To make things more complicated, there were attempts to interpret dissipation and entropy in kinetic-molecular context (see [10,11,12,13,14,15]). Maxwell and Thomson understood dissipation as the transformation of macroscopic ordered motion into chaotic motion at the molecular scale, and they considered it a matter of probability. In Austria, Boltzmann gave various statistico-mechanical entropy formulas starting in 1871, and by 1877 (at least) he regarded entropy increase as highly probable only. For the following, it will be good to remember that in the 1860s and 1870s thermodynamics was still a young, incompletely understood theory. Its basic concepts and methods were still in flux; its scope was not fully appreciated (especially in chemistry); and there were still dreams of perpetual motion of the second kind.

### 2.2. Loschmidt’s Columns of Salted Water (1869)

In 1869, a senior and yet newly appointed researcher in Vienna’s Physics Institute, Josef Loschmidt, remarked that the diffusion of a salt into water permitted the production of work from a single heat source, and discussed the compatibility of this fact with the second law of thermodynamics. After a failed industrial venture, Loschmidt had long been a school teacher with a passion for chemistry and physics. Largely self-taught but enjoying friendly support from the head of the Physics Institute, Josef Stefan, he contributed original work at the border between physics and chemistry, including his famous determination of the Avogadro-Loschmidt number in 1865 [16]. In his memoir of 1869 “On the second principle of thermodynamics” [17], Loschmidt described a thought-experiment with a valve letting only the faster molecules on one side of a wall pass to the other side, two years before Maxwell published his famous demon argument [18] (see [19]). Loschmidt’s aim was to shed doubts on “Clausius’s axiom”, according to which heat cannot be transferred from a colder to a warmer body without compensation. He nonetheless trusted the “second principle” according to which no work can be produced though a cyclic process involving a single heat source, enough so to study its thermochemical consequences.

In the main part of his memoir, Loschmidt considered a tall column of water at the bottom of which a large quantity of salt is introduced. The salt then dissolves into the water and slowly migrates to the top of the solution by diffusion. At the end of this process, the salt is globally higher than at the beginning and has thus worked against gravity even though the temperature is kept constant through the process. Loschmidt underlined that work was thus produced from a single heat source, although he did not confuse this possibility with a violation of the second law. In order to apply this law, he imagined the following cycle of reversible operations: the salt first migrates upwards by diffusion; a fixed quantity of the solution is then extracted from the upper layers of the column; this solution is evaporated to separate the salt; the vapor is then condensed back into the solution at the top of the column; and the separated salt is added to the bottom of the column. By the second law, he required the vanishing of the net work done in this cycle, and thus obtained a relation between the vapor pressure on top of the column and the chemical affinity of the salt with water. He also proved that in the equilibrium state the water column could not be saturated at its top.

Loschmidt actually built water columns in the basement of the Physics Institute to test the latter prediction, although he gave up after realizing that the diffusion was much too slow for equilibrium to be reached in a reasonable amount of time. In the same year, he discussed his valve-based violation of Clausius’s axiom with Stefan and his junior colleague Boltzmann. The latter countered that no intelligent being could exist and operate the valve in a strictly monothermal cellar. To which Stefan humorously commented: “Then I understand why your [Loschmidt’s] experiments with tall glass tubes in the basement have so miserably failed” (see [20], p. 231) Whatever be the true cause of Loschmidt’s failure to concretize his thought experiments, he recognized that the production of work by diffusion played an important role in chemical reactions involving the diffusion of a substance into another. He thus pioneered a basic idea of chemical thermodynamics.

### 2.3. Horstmann’s Dissociation Theory (1873)

Loschmidt’s insights went mostly unnoticed. In 1873, August Heinrich Horstmann [21], a Heidelberg chemist and former student of Gustav Kirchhoff and Hermann Helmholtz, approached the theory of chemical dissociation by means of Clausius’s concepts of entropy and disgregation (see [22,23]). In a dissociation equilibrium, Horstmann proposed, the dissociation degree has to take the value for which the entropy of the system is a maximum. Implicitly, by “system” he meant the mixture of the various reactants plus the thermostat. Calling $S_{x}$ the entropy of the mixture for the value *x* of the dissociation degree and $H_{x}=x\Delta H$ the heat thereby received from the thermostat at constant pressure, Horstmann’s equilibrium condition gives

(5) $$\frac{\partial }{\partial x}\left(-\frac{H_{x}}{T}+S_{x}\right)=0.$$

This is equivalent with the condition $\partial G_{x}/\partial x=0$ that we would now write in terms of the free enthalpy $G_{x}$ of the mixture.

Horstmann believed that the only contribution to the variation of the entropy $S_{x}$ was the variation of the corresponding disgregation $Y_{x}$. This is why instead of the former equation, he wrote

(6) $$-\frac{\Delta H}{T}+\frac{\partial Y_{x}}{\partial x}=0$$

(in a different notation). According to Clausius’s idea of disgregation as the degree of dispersion of the molecules, in the case of gases the disgregation of each component of the mixture should be computed as if it were alone in the container. This implies

(7) $$Y_{x}=\sum_{i}n_{i}(x)y_{i},$$

wherein $n_{i}$ is the number of moles of the component *i* and $y_{i}$ is the disgregation of a mole of the component at the partial pressure $P_{i}$. For a chemical reaction with the stoichometric coefficients $a_{i}$ (for the reaction ${2H}_{2}O↔{2H}_{2}+O_{2}$, the coefficients are $a_{1}=-2$, $a_{2}=2$, and $a_{3}=1$), we have $dn_{i}=a_{i}dx$ so that condition (6) reduces to

(8) $$\frac{-\Delta H}{T}+\sum_{i}a_{i}y_{i}=0$$

(owing to homogeneity, we have $\sum_{i}a_{i}\partial y_{i}/\partial x)=0$). For a perfect gas, Clausius’s definition of disgregation leads to

(9) $$y_{i}=y_{i}{}^{0}-R\ln P_{i},$$

wherein $y_{i}{}^{0}$ denotes the disgregation of the gas at the standard pressure. Combining with the previous equation, this gives

(10) $$\prod_{i}P_{i}{}^{a_{i}}=K,\;\mathrm{with}\;\Delta H-T\Delta Y+RT\ln K=0.$$

Horstmann thus obtained the dissociation law for a reaction involving gases only, with a slightly erroneous expression of the equilibrium constant *K*. His result agrees with the predictions of modern thermochemistry, except that the disgregation *Y* should be replaced with the entropy *S*.

The former considerations apply to reactions between gases, for instance the dissociation equilibrium of steam. Horstmann also treated the dissociation of solid substances (for instance calcium carbonate) and the dissociation of a salt into water (dissolution). In the first case, the molar disgregation of the solid components intuitively does not depend on their quantity, which implies that the equilibrium constant involves only the pressure of the gas components (for instance, the pressure of the carbon dioxide must be a temperature-dependent constant in the dissociation equilibrium ${\mathrm{CaCO}}_{3}↔\mathrm{CaO}+{\mathrm{CO}}_{2}$ recently studied by Henry Debray). In the second case, Horstmann assumed that the disgregation of the solutes varied with their concentration in analogy with the gas case. In all cases, he found his laws well confirmed by the already numerous empirical studies of dissociation equilibrium.

Despite the archaic reliance on disgregation, despite the confusion between this quantity and entropy, and despite the imprecise character of Horstmann’s reasoning (he did not clearly define the relevant systems and constraints), his memoir has often been regarded as inaugurating modern thermochemistry. This is well deserved, for Horstmann there derived chemical equilibrium from a maximum condition for the entropy of a properly defined system and made this equilibrium depend on the competition between heat and entropy production during the reaction.

### 2.4. Rayleigh on Dissipation and Diffusion (1875)

With Loschmidt’s and Horstmann’s exceptions, no one tried to apply thermodynamics to chemical processes until 1875. Possible reasons for this neglect were the aforementioned avoidance of irreversible processes, the empirical complexity of the conditions of most chemical reactions, and the common belief that the possibility of a chemical reaction depended on the development of heat (Thomsen-Berthelot principle). But there were obvious exceptions to this principle (endothermic reactions) and, as was just mentioned, there was a growing number of empirical laws in need of a theoretical explanation for various kinds of chemical equilibrium (see [22]).

In Britain, Lord Rayleigh was first, in 1875, to deplore the neglect of chemical thermodynamics in a lecture [24] he gave at the Royal Institution on the dissipation of energy. In Thomson’s understanding of the second law of thermodynamics, the energy originally available in a system for the production of work can be “dissipated”. For instance, some of the work transmitted by a mechanical machine can be lost by friction, or the work produced by a Carnot engine can be lost if the heat from the hot source directly goes to the cold source instead of acting on the engine. In the first case, dissipation corresponds to the creation of heat, in the second to its conduction. In 1874, Thomson [25] also cited the interdiffusion of two gases as a dissipative process in kinetic-molecular analogy with heat conduction. He did not address chemical reactions.

In contrast, in the following year Rayleigh stated [24] (p. 388):

The chemical bearings of the theory of dissipation are very important, but have not hitherto received much attention. A chemical transformation is impossible, if its occurrence would involve the opposite of dissipation.

Rayleigh thus understood, independently of Horstmann, that dissipation rather than heat development, determined the possibility of a chemical reaction. As a simple counter-example of Berthelot’s principle, he cited the dissolution of a salt into water. The dissolution could occur despite its endothermic character because the dissolution involved dissipation that could compensate for the cooling of the solution. In order to prove dissipation in ordinary dissolution, Rayleigh imagined a reversible process of dissolution in which work would be produced: the water is placed under a piston in a cylinder maintained at constant temperature; the piston is slowly raised until the water is fully evaporated and its pressure reaches the value below which it ceases to be absorbable by the salt; the salt is then brought into contact with the vapor; the piston is slowly pushed down until the vapor is fully condensed. The work the piston produces during its rise is larger than the work it receives during its descent because the pressure is smaller in the latter process. Therefore, work is gained during the global process. Ordinary, irreversible dissolution is dissipative because it implies the loss of an opportunity to produce work. Rayleigh went on to deplore the difficulty of applying thermodynamic principles to chemistry, because in general chemical processes were irreversible. At the same time, he noted the possibility of displacing a dissociation equilibrium in a reversible manner as Henry Debray had recently done in the case of calcium carbonate.

**Figure 2:**
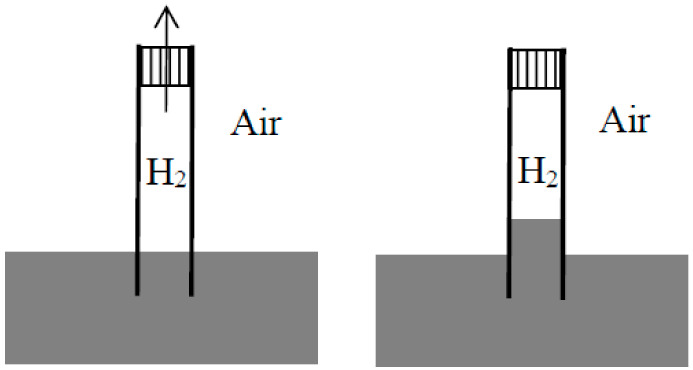
The monothermal elevation of water by diffusion according to Rayleigh.

Having understood that ordinary dissolution implied dissipation and having showed how to quantify this dissipation in a thought experiment, Rayleigh did the same for the mixing of two gases in a remarkable article [26] published a little later in the *Philosophical magazine*. In his brief introduction, he described a “common experiment” in which a tube was filled with hydrogen, closed at its upper end with a porous plug of Paris plaster, and immersed at its lowered end in water. Owing to the diffusion of the hydrogen through the plug into the air, the pressure diminishes in the tube and the water rises in the tube (see Figure 2). The heat from a monothermal environment is thus turned into work. Rayleigh went on [26] (p. 311):

Whenever then two gases are allowed to mix without the performance of work, there is dissipation of energy, and an opportunity of doing work at the expense of low temperature heat has been for ever lost. The present paper is an attempt to calculate this amount of work.

Rayleigh then announced the result of this calculation for chemically inactive gases obeying Dalton’s law: the maximal work that can be obtained by mixing (isothermically) two gases initially occupying the separate volumes $V_{1}$ and $V_{2}$ and finally sharing the volume $V_{1}+V_{2}$ is equal to the work produced by the expansion of the first gas alone from $V_{1}$ to $V_{1}+V_{2}$ plus the work produced by the expansion of the second gas alone from $V_{2}$ to $V_{1}+V_{2}$. The maximal work is reached when the mixing is reversible.

Rayleigh imagined three ways of reversible mixing or reversible separation, the first based on the condensation of one of the gases, the second on its chemical absorption, and the third on the differential action of gravity on the two gases. The result must be the same whatever the means of separation, for in the contrary case it would be possible to produce work in a monothermal cycle of operations.

**Figure 3:**
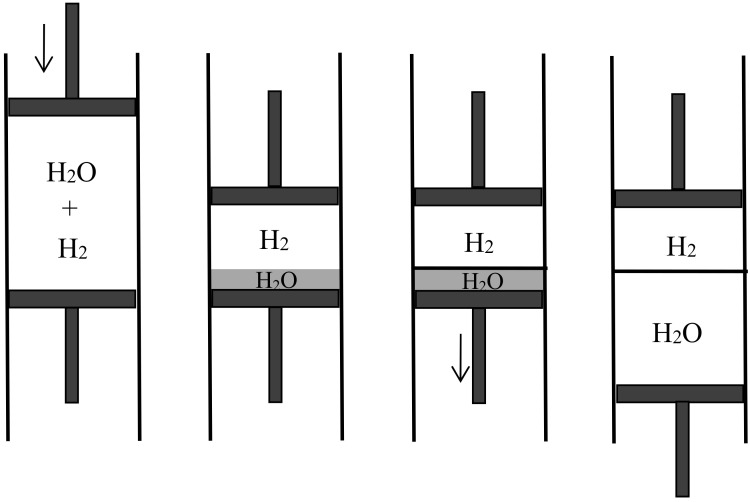
The successive steps of Rayleigh’s procedure for the reversible, isothermal separation of a mixture of steam and hydrogen.

First assume that one of the gases can be condensed, as is the case for a mixture of steam and hydrogen (see Figure 3). Compress the mixture isothermically in a piston until the steam is (almost) completely condensed. Then separate the condensed water from the hydrogen and evaporate it isothermically in a piston until the sum of the volume $V_{1}$ of the hydrogen and of the volume $V_{2}$ of the steam becomes equal to the volume of the original mixture. By Dalton’s law, the pressure in the mixture is the sum of the partial pressures of its components. Consequently, the net work done on the system during the reversible separation is equal to the work needed to compress the hydrogen from $V_{1}+V_{2}$ to $V_{1}$ and the vapor from $V_{1}+V_{2}$ to $V_{2}$, in conformity with the general result announced by Rayleigh.

**Figure 4:**
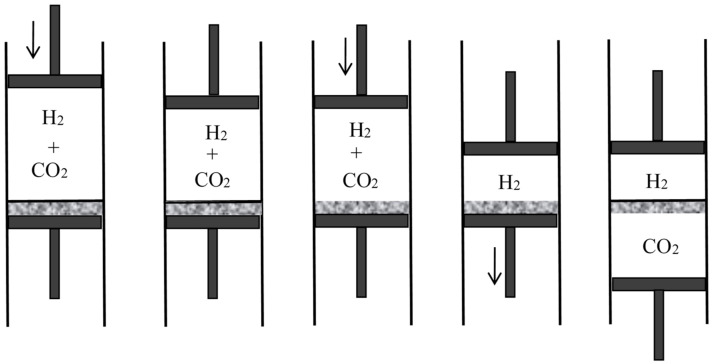
The successive steps of Rayleigh’s procedure for the reversible, isothermal separation of a mixture of hydrogen and carbon dioxide. The marbled area represents calcium oxide (CaO) in the two first steps, a mixture of calcium oxide and calcium carbonate (CaCO_3_) in the third and fourth steps, and again pure calcium oxide in the final state.

In Rayleigh’s second reasoning, the mixture is made of carbon dioxide and hydrogen (see Figure 4). The volume $V_{1}+V_{2}$ of the mixture is decreased slowly and isothermically to the value $V_{1}$ for which the partial pressure of the carbon dioxide reaches its equilibrium value in presence of calcium carbonate at the given temperature (this value is well-defined by Debray’s law). During further slow isothermic compression of the mixture in the presence of calcium oxide, the pressure of the carbon dioxide remains constant and it is gradually absorbed by the calcium oxide. Once the absorption is nearly complete, the hydrogen and the calcium carbonate are separated. The volume over the calcium carbonate is increased until it reaches the value $V_{1}$, and the hydrogen is expanded to the volume $V_{2}$.

**Figure 5:**
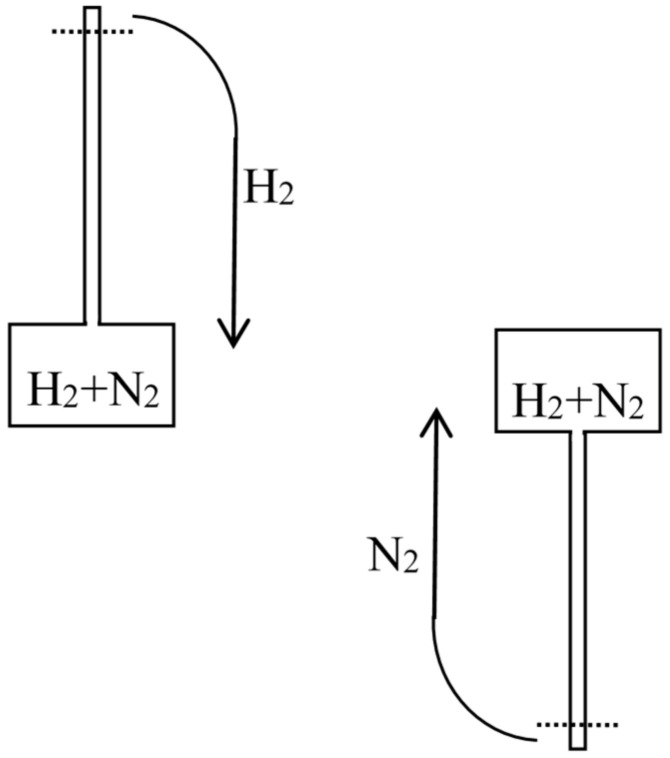
Schematic representation of Rayleigh’s method for the separation of two gases of different atomic weight (here hydrogen and nitrogen) by gravity.

In the third reasoning, the two mixed gases are perfect gases of different density and their separation is achieved by gravity (see Figure 5). For this purpose, the mixture is placed in a large container on which a long narrow vertical tube (closed on top) is mounted. Applying the laws of aerostatics to the lighter component, its pressure near the top of the tube is given by $P_{1}\prime =P_{1}e^{-k_{1}gh}$, in which $P_{1}$ is the partial pressure in the container, *h* the height of the tube, *g* the acceleration of gravity, and $k_{1}$ the proportionality coefficient between pressure and density. The height *h* is supposed to be so large that the pressure of the heavier component is there negligible. Remove a small volume $\upsilon_{1}\prime$ of this gravity-separated gas, let it fall to the level of the container, and compress it to the pressure $P=P_{1}+P_{2}$ in the container. The removal does not imply any work nor any change in the container if it is done after walling off the portion from the rest of the gas mixture (I add this condition to simplify Rayleigh’s reasoning). The fall produces the work $m_{1}gh$ with $m_{1}=\rho_{1}\prime \upsilon_{1}\prime =k_{1}P_{1}\prime \upsilon_{1}\prime$. The compression requires the work $P_{1}\prime \upsilon_{1}\prime \ln(P/P_{1}\prime )$ (for a perfect gas). Taking into account $P_{1}\prime \upsilon_{1}\prime =P\upsilon_{1}$ and $\ln(P_{1}/P_{1}\prime )=k_{1}gh$, the net work needed for the reversible, isothermal separation of the volume $\upsilon_{1}$ of the first gas from a large amount of the mixture at the same total pressure is

(11) $$w_{1}=P_{1}\prime \upsilon_{1}\prime \ln(P/P_{1}\prime )-m_{1}gh=P\upsilon_{1}\ln(P/P_{1}).$$

The similar separation of the volume $\upsilon_{2}$ of the second gas (by means of a downward tube) requires the work $P\upsilon_{2}\ln(P/P_{2})$. These two separations require the same work as the complete reversible separation of the volume $\upsilon_{1}+\upsilon_{2}$ of the mixture. The work needed for the latter separation therefore is

(12) $$w=P\upsilon_{1}\ln\frac{\upsilon_{1}+\upsilon_{2}}{\upsilon_{1}}+P\upsilon_{2}\ln\frac{\upsilon_{1}+\upsilon_{2}}{\upsilon_{1}},$$

in conformity with Rayleigh’s general rule (Schrödinger [27] rediscovered Rayleigh’s procedure in 1921 as a means for the reversible separation of isotopes).

To sum up, in 1875 Rayleigh proved that the interdiffusion of two gases implied dissipation, that is, the loss of an opportunity to produce work. He quantified the dissipation by determining the work produced in a reversible process connecting the mixed state to the unmixed state. He found this work to be the one given by the separate expansion of each gas from the initial to the final available volume. He did not comment on the evident fact that for perfect gases the value of this work does not depend on the nature of the two gases, as long as they are different.

## 3. Gibbs on the Equilibrium of Heterogeneous Substances

### 3.1. The Rules of Equilibrium (1873–1876)

In 1875, Josiah Willard Gibbs was in his fourth year on the chair of mathematical physics at Yale university, from which he held an engineering degree. In earlier years he had attended the lectures of a few famous mathematicians and physicists in France and in Germany; his interest then seems to have been mostly in optics and electrodynamics. It is not clear why, in the early 1870s, he decided to concentrate on thermodynamics. The trigger may have been his reading of Tait’s *Sketch of thermodynamics* in 1868, and Maxwell’s *Theory of heat* in 1871, as well as his witnessing lively debates in the *Philosophical magazine* about priority for the central concepts of the theory and about the meaning, proper definition, and usefulness of Clausius’s entropy concept of 1865 (see [28], pp. 51n–52n). Tait and Maxwell favored Thomson’s dissipation and reinterpreted or misinterpreted entropy in terms of the more practical concept of available energy.

In contrast with British natural philosophers, Gibbs welcomed Clausius’s entropy and perceived its potential for a more rigorous, systematic, and mathematical approach to thermodynamics. In his two first memoirs [28,29], published in 1873, he introduced entropy-temperature and entropy-volume diagrams to describe the properties of homogeneous substances including the recently discovered critical point. He also described and exploited the properties of the entropy surface in (S,P,T)-space. In particular, he discussed the equilibrium and stability of a given state of a substance under fixed pressure and temperature by means of the condition that the thermodynamic function U+PV−ST (our free enthalpy) should be a minimum [28] (p. 43n). He thereby assumed that volume, energy, and entropy were extensive quantities; and that, for a non-equilibrium state, they were still well-defined for the smaller parts of the substance: “The body, however, as a whole has a certain volume, entropy, and energy, which are equal to the sums of the volumes, etc., of its parts” [28] (p. 39).

Gibbs soon extended his entropy-based approach to chemical equilibrium in a book-size memoir “On the equilibrium of heterogeneous substances,” published between 1875 and 1878 in the *Transactions* of the Connecticut Academy [30]. His condition of stable equilibrium for a closed system was that the entropy should be a maximum at constant energy, or, equivalently, that the energy should be a minimum at constant entropy. For a homogeneous part of the system (that is, of uniform chemical composition and physical state), the energy *U* is a function of the temperature *T*, the volume *V*, and the number of moles $n_{i}$ of the various chemical components, and its differential is given by

(13) $$dU=TdS-PdV+\sum_{i}\mu_{i}dn_{i},$$

in which *P* is the pressure and the $\mu_{i}$ coefficients are the “chemical potentials.” For the equilibrium of a system made of two homogeneous and chemically stable parts (“phases”), Gibbs’s criterion gives

(14) $$T\prime dS\prime +T\prime \prime dS\prime \prime -P\prime dV\prime -P\prime \prime dV\prime \prime +\sum_{i}\mu_{i}\text{'}dn_{i}\prime +\sum_{i}\mu_{i}\text{"}dn_{i}\prime \prime =0$$

for any change of the variables such that

(15) $$dS\prime +dS\prime \prime =0,\;\;dV\prime +dV\prime \prime =0,\;\;dn_{i}\prime +dn_{i}\prime \prime =0.$$

This yields T′=T″, P′=P″,$\mu_{i}\prime =\mu_{i}\prime \prime$: for each chemical component present in the two phases, the temperatures, pressures, and chemical potentials must have the same value in the two phases. Another simple case is that of a homogeneous chemical mixture, whose proportion may vary by a chemical reaction of stoichiometric coefficients $a_{i}$(for ${2H}_{2}O↔{2H}_{2}+O_{2}$, $a_{1}=-2$, $a_{2}=2$, and $a_{3}=1$). Here equilibrium requires

(16) $$\sum_{i}\mu_{i}dn_{i}=0\;\;\mathrm{whenever}\;\;dn_{i}=a_{i}dx.$$

This yields

(17) $$\sum_{i}a_{i}\mu_{i}=0$$

as the effective condition of chemical equilibrium (Gibbs 1875–1878).

Gibbs also introduced the functions F=U−TS and G=U+PV−TS, such that

(18) $$dF=-SdT-PdV+\sum_{i}\mu_{i}dn_{i}$$

and

(19) $$dG=-SdT+VdP+\sum_{i}\mu_{i}dn_{i},$$

and which may be used instead of the energy in expressing the equilibrium condition when temperature equilibrium and pressure equilibrium have already obtained respectively. He regards the extensivity of the energy and the entropy as obvious [30] (p. 141) (I use the now standard letters for the thermodynamic potentials, not Gibbs’s):

We know, . . . a priori, that if the quantity of any homogeneous mass containing *s* independently variable components varies and not its nature or state, the quantities [*U*, *S*, *V*, $n_{1},n_{2},\ldots ,n_{s}$] will all vary in the same proportion.

Consequently, the function $G(T,P,n_{1},n_{2},\ldots )$ is a homogenous function of degree one in the variables $n_{i}$ and it therefore satisfies

(20) $$G=\sum_{i}n_{i}\frac{\partial G}{\partial n_{i}}=\sum_{i}n_{i}\mu_{i}.$$

In order to deal with chemical reactions between gases, Gibbs needed to determine the thermodynamic properties of a mixture of gases. For this purpose, he relied on the following empirical law [30] (p. 215):

If several liquid or solid substances which yield different gases or vapors are simultaneously in equilibrium with a mixture of these gases (cases of chemical actions between the gases being excluded), the pressure in the gas mixture is equal to the sum of the pressures of the gases yielded at the same temperature by the various liquid or solid substances taken separately.

This law crucially involves the possibility of separately condensing the various components of the gas mixture. Since the chemical potentials in the condensed phases are generally unaffected by the existence of other substances, it further implies that “The pressure in a mixture of different gases is equal to the sum of the pressures of the different gases as existing each by itself at the same temperature and *with the same value of its potential*”(emphasis mine)*.* This should not be confused with Dalton’s law, which simply states that the pressure in a mixture of different gases is equal to the sum of the pressures of the different gases as existing each by itself at the same temperature and *within the same volume as the volume of the mixture.* The manner in which Gibbs exploits his pressure rule is highly ingenious [30] (pp. 218–219).

In symbols, this pressure rule gives him

(21) $$P(T,\mu_{1},\mu_{2},\ldots \mu_{s})=\sum_{i}P_{i}(T,\mu_{i})\;\;\mathrm{and}\;\;dP(T,\mu_{1},\mu_{2},\ldots \mu_{s})=\sum_{i}dP_{i}(T,\mu_{i}).$$

Gibbs also has Equations (19) and (20),

$$G=\sum_{i}n_{i}\mu_{i}\;\;\mathrm{and}\;\;dG=-SdT+VdP+\sum_{i}\mu_{i}dn_{i},$$

which together imply

(22) $$dP(T,\mu_{1},\mu_{2},\ldots \mu_{s})=(S/V)dT+\sum_{i}(n_{i}/V)d\mu_{i}.$$

If the gas *i* were alone in the volume *V*, we would instead have

(23) $$dP_{i}(T,\mu_{i})=(S_{i}/V)dT+(n_{i}\prime /V)d\mu_{i}.$$

Combining Equations (21)–(23), we first get $n_{i}=n_{i}\prime$, which means that the number of moles of a given component of the mixture is the same as the number of moles of this gas when it occupies alone the volume *V* with the same chemical potential as in the mixture. Secondly, we get the mixing rules

(24) $$P(T,V,n_{1},n_{2},\ldots n_{s})=\sum_{i}P_{i}(T,V,n_{i})\;\;\mathrm{and}\;\;S(T,V,n_{1},n_{2},\ldots n_{s})=\sum_{i}S_{i}(T,V,n_{i}).$$

Similar rules apply to the functions *G*, *F*, *H*, and *U* thanks to the relations $G=\sum_{i}n_{i}\mu_{i}$, F=G−PV, H=G+TS. In modern words, the pressure, entropy, free enthalpy, free energy, enthalpy, and energy of the gas mixture is the sum of the corresponding quantities for each component as existing by itself at the same temperature and in the same volume. In Gibbs’s words [30] (p. 218),

The quantities [*P*, *S*, *U*, *F*, *G*, *H*] relating to the gas-mixture may therefore be regarded as consisting of parts which may be attributed to the several components in such a manner that between the parts of these quantities which are assigned to any component, the quantity of that component, the potential for that component, the temperature, and the volume, the same relations shall subsist as if that component existed separately. It is in this sense that we should understand the law of Dalton, that every gas is as a vacuum to every other gas.

Gibbs was aware of Rayleigh’s recent article on gas mixing, and he did not fail to note that Rayleigh’s rule for calculating the work produced by reversible isothermal mixing agreed with his own rule for combining free energies [30] (p. 221n). Indeed, this work is equal to the variation of the free energy, and by Gibbs’s rule the final free energy should be the same as if the two gases existed separately in the container.

### 3.2. The Gibbs Paradox (April 1876)

According to the previous rule, when two moles of two different perfect gases originally in two contiguous vessels of the same volume *V* are mixed by removing the wall between the two vessels, the entropy variation is the same as if each gas were expanded isothermally from *V* to 2*V*. It is therefore equal to 2Rln2. Gibbs comments:

It is noticeable that the value of this expression does not depend upon the kinds of gas which are concerned, if the quantities are such as has been supposed, except that the gases which are mixed must be of different kinds. If we should bring into contact two masses of the same kind of gas, they would also mix, but there would be no increase of entropy.

This is, in its rawest form, what we now call the Gibbs paradox because we intuitively expect a physical quantity to vary continuously during a continuous deformation of the system (when the difference in the kinds of the two gases goes to zero). Gibbs does not call this a paradox and goes on to explain why there is a mixing entropy for different gases and not for identical gases [30] (pp. 227–228) (The printing date “April 1876” appears on p. 217, “May 1876” on p. 233):

But in regard to the relation which this case bears to the preceding, we must bear in mind the following considerations. When we say that when two different gases mix by diffusion, as we have supposed, the energy of the whole remains constant, and the entropy receives a certain increase, we mean that the gases could be separated and brought to the same volume and temperature which they had at first by means of certain changes in external bodies, for example, by the passage of a certain amount of heat from a warmer to a colder body. But when we say that when two gas-masses of the same kind are mixed under similar circumstances there is no change of energy or entropy, we do not mean that the gases which have been mixed can be separated without change to external bodies. On the contrary, the separation of the gases is entirely impossible. We call the energy and entropy of the gas-masses when mixed the same as when they were unmixed, because we do not recognize any difference in the substance of the two masses.

Gibbs is here judging from a macroscopic, operational point of view: either the gases are different enough to allow entropy-decreasing separation, or their mixing does not imply any change of state. He next explains what counts as a change of state in thermodynamics:

So when gases of different kinds are mixed, if we ask what changes in external bodies are necessary to bring the system to its original state, we do not mean a state in which each particle shall occupy more or less exactly the same position as at some previous epoch, but only a state which shall be undistinguishable from the previous one in its sensible properties. It is to states of systems thus incompletely defined that the problems of thermodynamics relate.

These remarks agree with the extensivity of the entropy earlier admitted by Gibbs: no sensible property is altered when two portions of the same gas at equal temperature and pressure are allowed to communicate. At this point of the text we seem to be approaching a solution to the initial paradox based on an operational definition of thermodynamic states.

Gibbs still sees a difficulty [30] (pp. 228–229):

But if such considerations explain why the mixture of gas-masses of the same kind stands on a different footing from the mixture of gas-masses of different kinds, the fact is not less significant that the increase of entropy due to the mixture of gases of different kinds, in such a case as we have supposed, is independent of the nature of the gases.

Now we may without violence to the general laws of gases which are embodied in our equations suppose other gases to exist than such as actually do exist, and there does not appear to be any limit to the resemblance which there might be between two such kinds of gas. But the increase of entropy due to the mixing of given volumes of the gases at a given temperature and pressure would be independent of the degree of similarity or dissimilarity between them. We might also imagine the case of two gases which should be absolutely identical in all the properties (sensible and molecular) which come into play while they exist as gases either pure or mixed with each other, but which should differ in respect to the attractions between their atoms and the atoms of some other substances, and therefore in their tendency to combine with such substances. In the mixture of such gases by diffusion an increase of entropy would take place, although the process of mixture, dynamically considered, might be absolutely identical in its minutest details (even with respect to the precise path of each atom) with processes which might take place without any increase of entropy. In such respects, entropy stands strongly contrasted with energy.

Here we have the full-blown paradox, involving the limit of infinitely small difference between two gases, and the even stranger case of two gases whose molecules differ only by their interactions with the molecules of a third substance. In both cases, the entropy cannot possibly be a function of the molecular dynamics of the system of the two mixed gases only, in contrast with the energy. The analogy between energy and entropy, which originally inspired Gibbs’s equilibrium principle, turns out to be a risky one.

Gibbs ends his discussion with a suggestion for what makes entropy so special:

Again, when such gases [differing only through their interaction with a third substance] have been mixed, there is no more impossibility of the separation of the two kinds of molecules in virtue of their ordinary motions in the gaseous mass without any especial external influence, than there is of the separation of a homogeneous gas into the same two parts into which it has once been divided, after these have once been mixed. In other words, the impossibility of an uncompensated decrease of entropy seems to be reduced to improbability.

The logic of this often-cited remark seems to be as follows. First consider a homogeneous gas of 2*N* traceable molecules, and assume that the first *N* molecules are in a given half of the container at time zero. After a sufficiently long time, we intuitively expect the recurrence of this state of affair (this is obvious for small *N*). Since the evolution of the gas depends only on the mutual forces of its molecules, the same kind of recurrence extends to the case in which the first *N* molecules and the last *N* molecules interact differently with the molecules of another substance (in the absence of this substance). In the latter case, the recurrence is a process in which entropy first increases and then decreases to return to its initial value. Consequently, entropy decrease is not impossible in a closed system. It is just extremely improbable.

Gibbs clearly regarded this reasoning as a proper conclusion to his discussion of the mixing paradox. Yet it is hard to see how the probabilistic character of the entropy law truly connects to the Gibbs paradox. It would seem that the molecular picture directly suggests the possibility of the de-mixing of two different gases, just as much as it suggests the possibility of recurrence in the case of a homogeneous gas. Indeed, Thomson, Maxwell, and Boltzmann frequently used the molecular intuition of interdiffusion in order to justify the statistical character of the entropy law. Thomson did so in a communication to *Nature* of May 1874 [25], in which he discussed dissipation, Maxwell’s demon, and the probabilistic character of thermal equilibrium and mixing. He even computed the probability that in an air-filled vessel all the oxygen molecules be found in one part of the vessel and all the nitrogen molecules in the complementary part. Gibbs’s remark on the statistical character of entropy decrease probably echoed Thomson’s and Maxwell’s considerations. Their logical connection with the Gibbs paradox remains unclear (to me). In general, Gibbs’s discussion of his paradox confuses us as much as it illuminates us, for he conflates three different levels of analysis: the operational level, the level of abstract entropy, and the molecular level.

## 4. Diffusion and the Second Law

As seen, the Gibbs paradox arose in discussions about the thermodynamics of gas mixing, especially in chemical reactions. As we will now see, this topic remained active and controversial after Gibbs’s publication, and the resulting developments informed subsequent discussions of the paradox.

### 4.1. Maxwell on Diffusion (1877)

In 1877 Maxwell wrote the article “Diffusion” for the ninth, thoroughly revised edition of the *Encyclopaedia Britannica*. He was the obvious choice for this task since he had pioneered the kinetic-molecular theory of gas diffusion in two capital memoirs of 1860 and 1867. In the last section of his article, entitled “On processes by which the mixture and separation of fluids can be effected in a reversible manner” [31] (pp. 642–646), he briefly described the contents of Rayleigh’s relevant memoir of 1875, he related it to Gibbs’s more recent consideration of the mixing entropy, and he drew a broad philosophical conclusion on the meaning of entropy. Maxwell mentioned Rayleigh’s three ways of reversible mixing (by condensation, by chemical absorption, and by gravity) and the resulting rule for calculating the work produced during this process. He then explained how irreversible (isothermal) mixing equivalently led to an increase of Clausius’s entropy or to a dissipation of available work, the latter being the product of the former by the absolute temperature.

Lastly, Maxwell noted that the energy dissipated during the interdiffusion of two gases originally occupying the same volume at the same temperature and pressure had the constant value 2RTln2 independent of the nature of the two gases, and went on to confront this thermodynamic result with the kinetic-molecular intuition of the diffusion process [31] (p. 645):

Let us now suppose that we have in a vessel two separate portions of gas of equal volume, and at the same pressure and temperature, with a movable partition between them. If we remove the partition the agitation of the molecules will carry them from one side of the partition to the other in an irregular manner, till ultimately the two portions of gas will be thoroughly and uniformly mixed together. This motion of the molecules will take place whether the two gases are the same or different, that is to say, whether we can distinguish between the properties of the two gases or not.

If the two gases are such that we can separate them by a reversible process, then, as we have just shewn, we might gain a definite amount of work by allowing them to mix under certain conditions; and if we allow them to mix by ordinary diffusion, this amount of work is no longer available, but is dissipated forever. If, on the other hand, the two portions of gas are the same, then no work can be gained by mixing them, and no work is dissipated by allowing them to diffuse into each other.

It appears, therefore, that the process of diffusion does not involve dissipation of energy if the two gases are the same, but that it does if they can be separated from each other by a reversible process.

In this variant of the Gibbs paradox, there is a conflict between molecular intuition and the phenomenology of dissipation: According to molecular intuition, there seems to be no difference between the cases of different and identical gases; yet energy is dissipated in one case and not in the other.

Maxwell goes on [31] (pp. 645–646):

Now, when we say that two gases are the same, we mean that we cannot distinguish the one from the other by any known reaction. It is not probable, but it is possible, that two gases derived from different sources, but hitherto supposed to be the same, may hereafter be found to be different, and that a method may be discovered of separating them by a reversible process. If this should happen, the process of interdiffusion which we had formerly supposed not to be an instance of dissipation of energy would now be recognized as such an instance.

This may be regarded as a variant of Gibbs’s remark that entropy refers to “sensible properties” that condition our ability to concretely demonstrate heterogeneity. More original is Maxwell’s remark that our discovery of a heretofore unsuspected heterogeneity would lead us to revise our assessment of dissipation. This leads Maxwell to his famous conclusion regarding the meaning of dissipation [31] (pp. 645–646):

It follows from this that the idea of dissipation of energy depends on the extent of our knowledge. Available energy is energy which we can direct into any desired channel. Dissipated energy is energy which we cannot lay hold of and direct at pleasure, such as the energy of the confused agitation of molecules which we call heat. Now, confusion, like the correlative term order, is not a property of material things in themselves, but only in relation to the mind which perceives them. A memorandum-book does not, provided it is neatly written, appear confused to an illiterate person, or to the owner who understands it thoroughly, but to any other person able to read it appears to be inextricably confused. Similarly the notion of dissipated energy could not occur to a being who could not turn any of the energies of nature to his own account, or to one who could trace the motion of every molecule and seize it at the right moment. It is only to a being in the intermediate stage, who can lay hold of some forms of energy while others elude his grasp that energy appears to be passing inevitably from the available to the dissipated state.

So for Maxwell, dissipation is in some sense subjective: it depends not only on the kinetic-molecular state of the system but also on the extent to which we can physically act on this state. For a demon “who could trace the motion of every molecule and seize it at the right moment” there never is any dissipation. For a human being who has a limited ability to perceive and control heterogeneities, dissipation depends on this ability and need be reassessed when this ability evolves. This differs from Gibbs’s conclusion that entropy increase is a matter of probability, although Maxwell had earlier insisted on the statistical nature of the second law and himself related it to the kinetic-theoretical understanding of diffusion in other texts [32] (see [33], pp. 604–605).

### 4.2. Preston’s Violation of the Second Law (1877)

**Figure 6:**
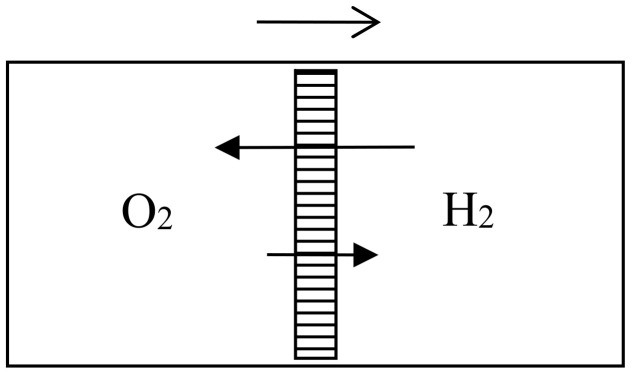
Preston’s device for the monothermal production of work by diffusion.

In the 1870s, Gibbs’s extraordinarily deep and thorough study of the laws of chemical equilibrium was known only to the happy few who, like Maxwell, privately received offprints from the Yale professor. The engineer-physicist Samuel Tolver Preston was not among them. More surprisingly, he does not seem to have read Rayleigh’s article on diffusion in the *Philosophical magazine*. This oversight allowed him, in a letter to *Nature* [34], to announce “an exception to the second law of thermodynamics” based on the different velocities with which two different gases diffuse through a porous wall. Specifically, he imagined a cylinder with a porous piston in the middle, oxygen gas on the left side, and hydrogen gas on the right side of the piston (see Figure 6). The mass of the hydrogen molecules being much smaller than that of the oxygen molecules, their average velocity much be much larger by energy equipartition. Consequently, the diffusion of the hydrogen into the oxygen is much faster than the opposite process and the pressure on the oxygen’s side must increase. The piston is thus able to perform work even though the entire system is originally at uniform temperature.

Preston also noted, as Loschmidt and Rayleigh had earlier done, that work could be generated by the differential diffusion of two gases of different density in a gravitational field. In general, he concluded that work could be obtained without a temperature difference, if only a density difference (of a diffusing gas) existed [34] (p. 32). Unlike his predecessors, he believed such processes to be violating the second law, and he even hoped they could prevent the heat death with which Thomson had threatened the entire universe. This belief is easily explained by his relying on Thomson’s early, rough statement of the second law as “the impossibility of producing work by cooling any portion of matter below the temperature of the coldest of the surrounding objects” [4] (p. 179). In reality, Thomson meant the impossibility of a monothermal engine that would be able to turn the heat form a single source into work *after completing a cycle of operations.* Preston simply overlooked the necessity of a cycle.

**Figure 7:**
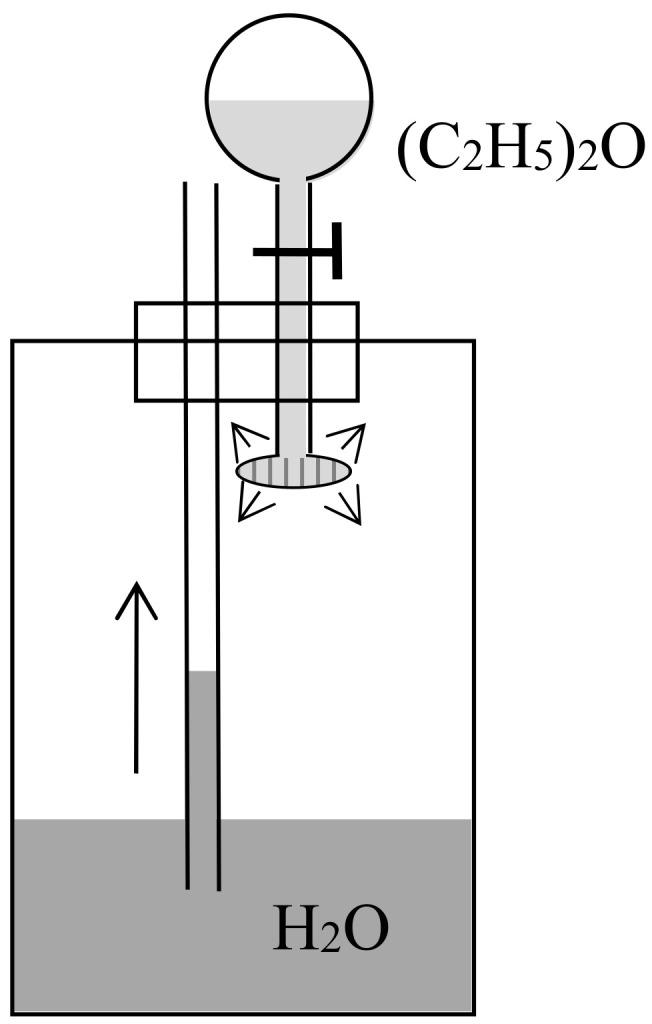
Aitken’s device for pumping up water by means of the diffusion of ether into air.

Preston persisted a few months later in a second letter to *Nature* [35] in which he prided himself on having found a concrete version of Maxwell’s demon. He now illustrated diffusion through a porous piston by a hard-ball gas model, and he perfected his imaginary device by allowing the periodic refilling of the two compartments of his cylinder with fresh hydrogen and oxygen. He also noted that the porous piston, instead of doing work through an external mechanical contraption, could move freely in the cylinder and thus heat up the gas on one side by compression and cool down the gas on the other side by expansion, in contradiction with Clausius’s statement of the second law (according to which heat cannot spontaneously pass from a colder to a warmer body). In the following issues of *Nature* he got only compliments: firstly by the future meteorologist John Aitken [36], who described a device that could elevate water through the diffusion of ether into air (see Figure 7); secondly by the astronomer Alexander Stewart Herschel [37] (the third of the dynasty) who imagined an obscure connection with Clausius’s virial.

The first public rebuttal occurred in the German *Annalen*, and it came from Clausius himself [38]. After praising Preston’s “ingenious considerations” and his “interesting conclusions,” Clausius denied that they implied any violation of the second law because a full and exact cycle of operations would be needed for that purpose. At the end of Preston’s pseudo-cycle (after refreshing the gases in the cylinder) not only heat has been borrowed from the environment but also an unmixed store of hydrogen and oxygen has been turned into a mixture. The mixing compensates for the monothermal production of work, as Loschmidt, Thomson, Rayleigh, and Gibbs clearly understood.

Presumably after seen Clausius’s note (he does not mention it) (Clausius’s note was written in May 1878, and Preston’s letter was published on 23 May 1878), Preston sent a third letter to *Nature* in which he now admitted that “the case there dealt with does not appear necessarily to be out of harmony with what is termed the ‘second law of thermodynamics,’ though it may be questioned whether it quite harmonises with certain modes of stating the law” [39] (p. 92). Just like Clausius, he now emphasized that the monothermal production of work, or the spontaneous transfer of heat from a colder to a warmer body, was accompanied with mixing in the environment. He nevertheless insisted about the practical promises of the possibility of producing work from a given source of heat without the need of a warmer or colder source.

### 4.3. Boltzmann on the Mixing Entropy (1878)

In the summer of 1878, Boltzmann became aware of Preston’s heresy through an abstract in the *Beiblätter*. His first reaction appeared in the November issue of the *Philosophical magazine* [40], together with an abstract of a recent memoir of his. In agreement with Clausius, he first noted that the production of work by diffusion did not violate the second law and rather was an interesting illustration of this law. Apparently unaware of Rayleigh’s and Gibbs’s publications on this topic, he then offered a new, kinetic-theoretical derivation of the mixing rule for entropies. His main resource was the combinatorial expression he had given in the previous year for the entropy of a homogeneous gas and also for a mixture of several gases [41]. Let us first recall how he arrived at this formula.

For a single monatomic gas, Boltzmann divided up the (r,v)-space of a molecule into uniform cells of size ε and counted the number of distributions of the *N* molecules of the gas over the cells for which there were $N_{i}$ molecules in the cell labeled by the index *i*. This number is equal to the number

(25) $$W=\frac{N!}{\prod_{i}N_{i}!}$$

of permutations of the molecules that leave the content of the various cells invariant. Boltzmann took this number to be proportional to the probability of the distribution ${\left(N_{i}\right)}_{i}$ and he identified the equilibrium distribution with the distribution of maximum probability under the constraints

(26) $$\sum_{i}N_{i}=N$$

and

(27) $$\sum_{i}N_{i}(m\upsilon_{i}{}^{2}/2)=E$$

of constant total number *N* and constant total energy *E.* Assuming the cells to be large enough to contain a large number of molecules, we may use the approximation

(28) $$\ln W=N\ln N-\sum_{i}N_{i}\ln N_{i}.$$

Assuming the cells to be small enough so that the relative variation of the number $N_{i}$ between two consecutive cells becomes negligible, we may replace the discrete distribution ${\left(N_{i}\right)}_{i}$ with the continuous distribution $f(r,v)d^{3}rd^{3}\upsilon$. Boltzmann’s problem then is to find the distribution *f* for which

(29) $$H=\int f\ln f\;d^{3}rd^{3}\upsilon$$

is a minimum under the conditions

(30) $$\int f\;d^{3}rd^{3}\upsilon =N\;\;\mathrm{and}\;\;\int (m\upsilon^{2}/2)f\;d^{3}rd^{3}\upsilon =E.$$

By the method of Lagrange’s multipliers, the solution is

(31) $$f=\alpha e^{-\beta m\upsilon^{2}/2},\;\mathrm{with}\;\alpha =(N/V){\left(m\beta /2\pi \right)}^{3/2}\;\;\mathrm{and}\;\;\beta =(3/2)(N/E).$$

To this solution corresponds

(32) $$\begin{array}{c} \ln W\approx N\ln N-\int f\ln f\;d^{3}rd^{3}\upsilon -N\ln\varepsilon =-N\ln(\alpha /N)+\beta E-N\ln\varepsilon \\ =-(3/2)N\ln\beta +N\ln V+(3N/2)\ln(2\pi e/m)-N\ln\varepsilon . \end{array}$$

This is to be compared with the entropy of a perfect gas, given by

(33) $$dS=c_{V}dT/T+PdV/T=(3/2)nRdT/T+nRdV/V,$$

wherein $c_{V}$ denotes the specific heat at constant volume, *R* the constant of perfect gases, and *n* the number of moles of the gas. This differential expression agrees with

(34) $$S=k_{B}\ln W\;\mathrm{provided}\;\mathrm{that}\;Nk_{B}=nR\;\mathrm{and}\;\beta =1/k_{B}T.$$

Note that the logarithm of the permutability naturally gives a non-extensive expression of the entropy, since NlnV is not extensive. Boltzmann felt free to drop the ugly NlnN and Nlnε terms in the expression of lnW (in fact, he directly worked with $-\int f\ln fd^{3}rd^{3}\upsilon$). This gave him the extensive form

(35) $$S/k_{B}=(3/2)N\ln\beta +N\ln(V/N)+(3N/2)\ln(2\pi \text{​​ ​}e/m).$$

For a mixture of monatomic (perfect) gases, the distribution of the molecules of a given component in the cells of the corresponding phase-space is independent of the similar distribution for another component. In the case of two components only, call $N_{i}\prime$ and $N_{i}\prime \prime$ the corresponding distributions. The total permutability is simply given by

(36) $$W=\frac{N\prime !}{\prod_{i}N_{i}\prime !}\frac{N\prime \prime !}{\prod_{i}N_{i}\prime \prime !}.$$

Again, Boltzmann felt free to omit the N′lnN′ and N″lnN″ terms in lnW and got

(37) $$S=-k_{B}\int f\prime \ln f\prime \;d^{3}r\prime d^{3}\upsilon \prime -k_{B}\int f\prime \prime \ln f\prime \prime \;d^{3}r\prime \prime d^{3}\upsilon \prime \prime$$

for the entropy.

As Boltzmann emphasized in his response [40] to Preston, this formula implies that the entropy of the mixture is given by the entropy of the components as if each of them existed alone in the container. This is exactly the Gibbs-Rayleigh rule, now justified by kinetic-theoretical means. For the contributions of the two gas components to the entropy of the mixture, Boltzmann directly used the thermodynamic formulas

(38) S′=(3/2)n′RlnT+n′RlnV and S″=(3/2)n″RlnT+n″RlnV.

If the two gases originally were in two separate containers of volumes $V_{1}$ and $V_{2}$ such that $V_{1}+V_{2}=V$ at the same temperature *T* and at the same pressure $P=n\prime RT/V_{1}=n\prime \prime RT/V_{2}$, then the original entropy is $S_{1}+S_{2}$ with

(39) $$S_{1}=(3/2)n\prime R\ln T+n\prime R\ln V_{1}\;\mathrm{and}\;S_{2}=(3/2)n\prime R\ln T+n\prime R\ln V_{2}.$$

The mixing entropy is (I have corrected a slip in Boltzmann’s expression of *S*_m_)

(40) $$S_{m}=S\prime +S\prime \prime -S_{1}-S_{2}=n\prime R\ln\frac{V_{1}+V_{2}}{V_{1}}+n\prime \prime R\ln\frac{V_{1}+V_{2}}{V_{2}},$$

in conformity with Gibbs’s result. The corresponding work gained during isothermal reversible mixing is $TS_{m}$. Lastly, Boltzmann sketched a method of reversible mixing based on absorption of one of the gases by a solid chemical (for instance ${\mathrm{CO}}_{2}$ by CaO) just as in Rayleigh’s article of 1875.

A few months later, Boltzmann published a fuller account of his views in the proceedings of the Viennese Academy [42]. For the entropy of a perfect homogeneous gas, he now used the extensive form

(41) S=(3/2)nRlnT+nRln(V/n),

with concomitant alterations in the expressions of $S_{1}$, $S_{2}$, S′, S″. The net result for the mixing entropy is the same since the n′Rlnn′ and n″Rlnn″ corrections are the same in the initial and final states. Boltzmann then investigated three ways of obtaining the maximal amount of work in reversible, isothermal mixing: by chemical absorption (pp. 311–312), by gravity combined with chemical absorption (pp. 313–316), and by means of semipermeable walls (p. 317). In the first case, the reasoning was similar to Rayleigh’s, except that Boltzmann recognized the ideal character of the imagined gases and substances and considered more complex processes in which the maximal work could be reached with real gases and absorbers. In the third case, the reasoning was entirely new and it turned out to be highly influential.

### 4.4. Semipermeable Walls

**Figure 8:**
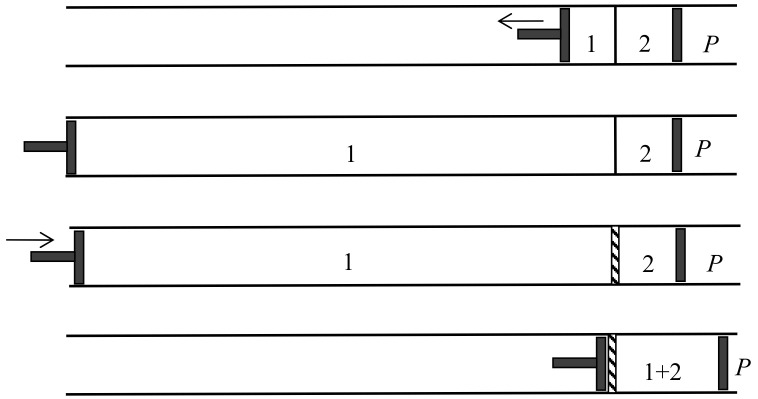
The successive steps of Boltzmann’s procedure for the reversible, isothermal mixing of two gases 1 and 2 by means of a semipermeable wall. The pressure *P* on the right side of the rightward sliding wall is kept constant. The hatched rectangle is permeable to gas 1 and impermeable to gas 2.

Semipermeable walls or diaphragms had long been known in relation to osmosis, in which the solvent is free to move across the diaphragm while the solute is confined on one side. They occurred in Gibbs’s memoir on the equilibrium of heterogeneous substances, as a particular case of equilibrium with osmosis in mind. No one, however, had imagined semipermeable diaphragms for gases and their application to reversible mixing. Rayleigh’s, Preston’s, and Aitken’s porous walls essentially differed from such diaphragms since they were permeable to the two gases being mixed; their purpose was to let the two gases permeate at different speeds. As Boltzmann noted, their way of mixing is irreversible and therefore cannot be used to produce the maximal work. In contrast, semipermeable walls allow reversible, isothermal mixing in a very simple manner. In the process imagined by Boltzmann [42] (p. 317) (see Figure 8), the two gases initially occupy separate containers of volumes $V_{1}$ and $V_{2}$ at the same pressure *P* and the same temperature *T*. The first gas is expanded slowly until its pressure is a vanishingly small fraction of *P*. This gas is then allowed to penetrate the second gas through a semipermeable diaphragm. The volume of its container is slowly reduced to zero while the volume of the second container is constantly adjusted to the value for which the total pressure remains *P*. At the end of this reversible process, the work produced is the work given by the isothermal expansion of the first gas from $V_{1}$ to $V_{1}+V_{2}$ plus the work given by the isothermal expansion of the second gas from $V_{2}$ to $V_{1}+V_{2}$. For perfect gases, this gives

(42) $$W=n\prime RT\ln\frac{V_{1}+V_{2}}{V_{1}}+n\prime \prime RT\ln\frac{V_{1}+V_{2}}{V_{2}}$$

if n′ and n″ denote the numbers of moles of the two gases. This formula agrees with the theoretical value $S_{m}/T$ of the maximal work that can be gained by mixing, $S_{m}$ being the mixing entropy of Equation (40). Plausibly, Boltzmann worked backwards from this formula to imagine the mixing by semipermeable walls.

There being no real semipermeable walls known to him for pairs of gases, Boltzmann called his semipermeable walls “fictional” and did not insist on them. Nonetheless, they soon became the principal ingredient of a standard derivation of the mixing entropy of two gases. In 1883, Hermann Helmholtz approved Rayleigh’s and Boltzmann’s considerations in a footnote (p. 654n) to the third installment [43] of his influential “Thermodynamik der chemischen Vorgänge,” based on the concept of “free energy.” In the same year his disciple Max Planck [44] dwelt on gas mixing and gas dissociation. He remarked (p. 370) that semipermeable walls allowed for reversible mixing and gave the example of glowing platinum, which is permeable to hydrogen and not to nitrogen.

**Figure 9:**
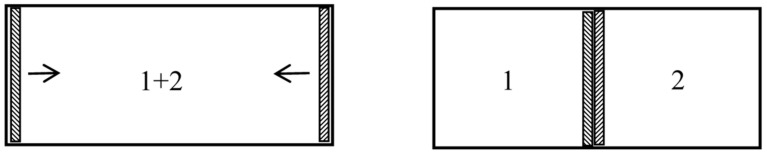
Carl Neumann’s procedure for the reversible, isothermal separation of two perfect gases 1 and 2 by displacing two semipermeable walls. One of the hatched walls is permeable to gas 1, the other to gas 2.

In 1891, the Leipzig mathematician and mathematical physicist Carl Neumann [45] (pp. 117–118) more explicitly considered a cylinder of volume 2V initially containing a mixture of two perfect gases and equipped with two sliding semipermeable pistons (see Figure 9). These pistons are slowly moved from the end sections of the cylinder to its middle section, so that in the end state the two gases are separated and each of them occupies the volume *V*. The net pressure on a given piston being equal to the partial pressure of the gas to which it is impermeable, and this pressure being inversely proportional to the volume occupied by this gas in an isothermal process, the network *W* done on the system is 2RTln2. The work conversely obtained by reversible mixing is given by the same formula. The mixing entropy then is

(43) $$S_{m}=\Delta S=Q/T=-W/T=2R\ln2,$$

in conformity with Gibbs’s formula. The mixing is here natural: the end state is the same as if the gases had diffused through each other by the mere removal of a partition.

**Figure 10:**
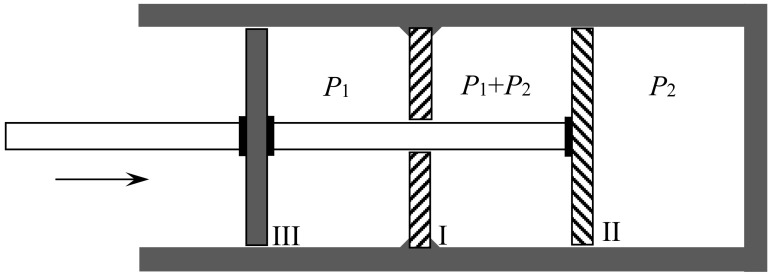
The isentropic, reversible mixing of two gases 1 and 2 by means of two semipermeable walls according to Planck.

In another kind of mixing, each of the two gases originally occupies a volume equal to the volume 2V of the end mixture. This mixing can be achieved reversibly and isothermally by first contracting each gas to the volume *V* and then mixing them according to the former procedure. The work done during the first process is exactly the opposite of the work done in the second. This mixing therefore does not alter the entropy. In other words, the entropy of an ideal mixture of two gases is the sum of the entropies that each gas would have if it occupied the available volume separately. This is Gibbs’s rule. In his *Grundriss der allgemeinen Thermochemie* of 1893 [46] (pp. 128–129), Planck recalled that Gibbs’s expression of the entropy of a gas mixture could be derived from the existence of semipermeable walls. In his lectures on thermodynamics of 1897 [47] (pp. 200–201), he described the reversible, isentropic mixing process that is now most commonly found in thermochemistry texts. In the cylinder and double piston of Figure 10, wall I is permeable only to the gas I at pressure $P_{1}$, wall II only to gas 2 at pressure at pressure $P_{2}$. The sliding walls II and III move together reversibly to the right without any expense of work since the forces on III and II have the same intensity and opposite directions. The temperature is constant and uniform throughout the process. At the end, the gases 1 and 2 have been mixed reversibly and isothermally, and the final volume is equal to the common volume of the two gases before mixing.

## 5. New Discussions of the Gibbs Paradox

### 5.1. Duhem and Neumann on Gas Mixtures (1886–1892)

In his lucid *Le potentiel chimique* of 1886 [48] (pp. 46–47), Pierre Duhem recognized the central character of Gibbs’s mixing rule and justified it as follows. The entropy variation of the mixture during an infinitesimal variation of its volume and temperature is given by

(44) $$dS=\frac{\delta Q}{T}=\frac{dU+PdV}{T}.$$

Owing to the relation $P=P_{1}+P_{2}$ between the total pressure and the partial pressures and to the relation $U=U_{1}+U_{2}$ between the total energy and the partial energies, we have

(45) $$dS=dS_{1}+dS_{2},\;\;\mathrm{with}\;\;dS_{1}=dU_{1}+P_{1}dV\;\;and\;\;dS_{2}=dU_{2}+P_{2}dV.$$

By integration, this implies that the entropy of the mixture is equal to the sum of the entropies that the components would have if they existed separately in the vessel, up to an additive constant independent of the temperature *T* and the volume *V* but possibly depending on the composition of the mixture (since the mass of each gas component is kept constant during the former infinitesimal variation). Duhem arrived at Gibbs’s rule by further excluding the latter dependence (Neumann [44] (p. 115) criticized Duhem for arbitrarily setting the integration constant to zero).

When, in 1892, Duhem published his treatise on gas dissociation [49], he had read Neumann’s memoir of 1891 [45] and his illuminating discussion of the mixing entropy. He now insisted (pp. 49–50), as Henri Poincaré had done in his Sorbonne lectures on thermodynamics [50] (pp. 320–321), that the former reasoning led to

(46) $$S=S_{1}+S_{2}+\psi (n_{1},n_{2}),$$

wherein the function $\psi (n_{1},n_{2})$ of the numbers of moles of the two components remains undetermined. An additional assumption was needed to reach Gibbs’s rule $S=S_{1}+S_{2}$. It could be equilibrium of the mixture with condensed phases according to Gibbs, equilibrium with a partially dissociated salt (calcium carbonate for a mixture of carbon dioxide with another gas), or the existence of semipermeable walls according to Neumann. In conformity with his general conception of physical theory, Duhem did not dwell on these justifications and rather regarded the extensivity of partial thermodynamic potentials and entropies as an axiom whose merit was to be judged on its empirical consequences.

In his impressive memoir of 1891 [45], Carl Neumann sought to clarify the foundations of thermodynamics and its variable applications, giving more precise statements of the basic assumptions, bringing implicit assumptions to light, strengthening the mathematical discussion, and testing the mutual compatibility of the various assumptions. He gave special attention to the mixing of gases and favored semipermeable walls as the simplest and most direct way to justify Gibbs’s mixing rule, although he also attended to reversible separation by gravity in Rayleigh’s manner (pp. 119–124). He noted that the resulting value of the maximal mixing work had received the stamp of Rayleigh’s and Helmholtz’s authority. Yet he expressed some “mistrust” in this result, for it seemed to have the absurd consequence that the mixing of two identical gases would produce the same amount of work. In his opinion, Gibbs’s discussion of this point “did not quite dissolve the present obscurity” (p. 129).

Duhem reacted to Neumann’s worries in his treatise of 1892 [49] (pp. 52–53):

In a recent and very important writing, a good part of it is devoted to the definition [of a gaseous mixture according to Gibbs], Mr. Carl Neumann points to a paradoxical consequence of this definition. This paradox, which must have stricken the mind of anyone interested in these questions and which, in particular, was examined by Mr. J. W. Gibbs, is the following:

If we apply the formulas relative to the mixture of two gases to the case when the two gases are identical, we may be driven to absurd consequences.

This is, as far as I know, the first occurrence of the word “paradox” in this context. Unlike Neumann, Duhem believed that Gibbs’s discussion, if properly sharpened, did solve the paradox. Duhem first recast Gibbs’s argument as the following syllogism:

- *Major premise*: The notion of mixture of two gases includes the mixture of two masses of the same gas.
- *Minor premise*: Gibbs’s definition of a mixture leads to absurd results when applied to the mixture of two masses of the same gas.
- *Conclusion*: Gibbs’s definition is inacceptable.

Duhem rejected the major premise: in his view, the notion of mixture applied only to *different* gases because in the case of two masses of the same gases, bringing them into contact did not imply any disequilibrium.

This reasoning indeed resembles the part of Gibbs’s discussion in which he brings forth that entropy should depend on states determined by sensible properties only. In Duhem’s philosophy, this is almost a tautology since physical theory in general and thermodynamics in particular can only refer to sensible properties. Atoms, molecules, and their motion are mere figments of the mind. As for Gibbs’s consideration of very nearly identical gases and the joint statement about the probabilistic nature of the entropy law, Duhem simply ignores them.

### 5.2. Wiedeburg’s “On Gibbs’ Paradox” (1894)

One of Duhem’s readers was Otto Wiedeburg, a Leipzig *Privatdozent* who had recently completed a PhD on hydrodiffusion in Berlin under August Kundt. In 1894, Wiedeburg [51] published an essay entitled “Das Gibbs’sche Paradoxon” in the *Annalen*, with proper reference to Gibbs’s, Neumann’s, and Duhem’s contributions. He first identified the two assumptions leading to the mixing entropy formula and paradox: (A) Each component of the mixture behaves as an ideal gas; (B) The pressure, energy, and entropy of the mixture are the sum of the contributions of each component regarded as alone in the container. He then gave his own statement of the paradox (pp. 684–685):

[The mixing entropy] as computed from these assumptions turns out to have a non-zero value independent of the nature of the gases. It would therefore have the same value when gas masses of the same chemical nature diffuse into each other. Yet one surely expects the value zero for the entropy variation, since there is no perceptible change in what is regarded as the ‘state of the system’ in thermodynamics and since entropy depends on this state only.

Gibbs, Wiedeburg went on, had tried to defuse the paradox by arguing an essential disparity between the case of different gases and the case of identical gases: in the latter case it is physically impossible to separate the two mixed gas masses, so that we lack the means to compute the mixing entropy. Duhem had similarly argued that bringing into contact two masses of the same gas at the same pressure and temperature did not imply any transformation. At that point of his text, Wiedeburg brought in the kinetic-molecular picture of the gases [51] (p. 685):

To this we may object that according to the kinetic intuition the inducement to mixing is equally given in both cases by the constant (though slow) migration of the smallest particles.

In other words, the kinetic-molecular intuition (implicitly admitting the distinguishability of the molecules or “smallest particles”) contradicts the extensivity of entropy. Wiedeburg, unlike Duhem and Planck, was taking the kinetic theory of gases seriously, presumably because his former advisor Kundt was one the few German promoters of this theory.

In order to shed light on this matter, Wiedeburg scrutinized Assumption B (Gibbs’s mixing rule). He approved Walther Nernst’s recent remark [52], in a review of the German translation of Gibbs’s thermodynamic papers, that this assumption should not be regarded as evident despite its formal appeal. The assumption, he went on, needed physical justification in Gibbs’s original manner (through equilibrium with condensed phases) or in Poincaré’s manner (based on the dissociation of calcium carbonate). He favored Neumann’s justification by means of semipermeable walls (bringing out the implicit assumption that the pressure of a mixture on a semipermeable wall is equal to the partial pressure of the gas to which it is permeable). In this concept he found his own solution to Gibbs’s paradox: there is no mixing entropy for two masses of the same gas simply because the concept of semi-permeability “presupposes a certain finite difference between the gases to be mixed” [51] (p. 693).

Wiedeburg next confronted this view with the kinetic-molecular intuition [51] (pp. 693–694):

However, if we admit the mental or even practical possibility to reversibly mix or unmix similar [gas] masses in such a way that every individually determined smallest particle is found in the same ‘state,’ in particular in the same position, after a complete cycle, it cannot be denied that in such a mixing process work can be won even though it does not involve any outward change. Simply, in this case the concept of ‘state of a system’ must be determined and handled in a much more extensive and precise manner than is usually done.

Wiedeburg here seems to make the definition of thermodynamic states relative to our ability to separately manipulate parts of the system. He may have had in mind the case of two gases whose molecules differ only through their interactions with the molecules of a third gas. Or he may have been alluding to Maxwell’s demon and to Maxwell’s later idea that dissipation occurs only for beings “in the intermediate stage”. Yet, toward the end of his article, he distanced himself from Gibbs’s probabilistic conclusion (p. 696):

It should be clear that one need not conclude, as Gibbs himself does, that ‘the impossibility of an uncompensated decrease of the entropy seems to be reduced to an improbability,’ in other words: that the second law of thermodynamics seems not to be absolutely true.

It should be remembered that the mid-1890s were the time of the most violent opposition between those, like Boltzmann, who regarded the kinetic molecular theory as the true foundation of thermal phenomena and reduced the second law of thermodynamic to a merely statistical law, and those, like Planck, who believed in a purely macroscopic thermodynamic based on two absolute laws. Wiedeburg prudently avoided to take side. He believed he could solve the Gibbs paradox without entering this debate, through an operational definition of thermodynamic states.

Wiedeburg still needed to address Gibbs’s consideration of the limit of a vanishing difference between two gases. He did so at the very end of his article [51] (p. 697):

The paradoxical consequences [of the mixing-entropy formula] start to occur only when we follow Gibbs in imagining gases that are infinitely little different from each other in every respect and thus conceive the case of identical gases as the continuous limit of the general case of different gases. On the contrary, we may well conclude that finite differences of the properties belong to the essence of what we call matter.

Planck echoed this view in his influential thermodynamics lectures of 1897 (without referring to Wiedeburg or even to Gibbs) [47] (pp. 203–204):

With regard to the entropy increase by diffusion, it does not make any difference whether the gases are chemically more or less ‘similar’ [*ähnlich*]. Now, if we take two identical gases, the entropy increase is obviously zero, since there is no change of state at all. Whence follows that the chemical difference between two gases or two substances in general, cannot be represented through a continuously variable quantity, and that we instead have to do with discrete distinctions [*sprungweisen Beziehungen*]: either equality [*Gleichheit*] or inequality [*Ungleichheit*]. This circumstance creates a principal opposition between chemical and physical properties, since the latter must always be regarded as continuously variable.

## 6. The *N*! Division

### 6.1. Boltzmann on Chemical Equilibrium (1883)

The Gibbs paradox results from the combination of two elements: the extensivity of entropy, and the Rayleigh-Gibbs rule for calculating the mixing entropy of two gases. The latter rule can be derived from the existence of a reversible mixing process, without presupposing the extensivity of entropy. To be true, Gibbs’s own derivation does appeal to extensivity since it relies on the relation $G=\sum_{i}n_{i}\mu_{i}$, which is itself a consequence of the homogeneity of the *G* function. But Boltzmann’s derivation does not (at least in its first form) necessitate extensivity. For two gases initially occupying the separate volumes $V_{1}$ and $V_{2}$ at the same temperature *T* and the same pressure *P*, the rule gives the mixing entropy as the sum of the entropy variations for each gas during reversible, isothermal expansion from the original volume to the final volume $V_{1}+V_{2}$. This rule derives from the existence of reversible mixing processes. Its application involves nothing more that the variation of the entropy function of a homogeneous gas for a constant value of the number of molecules. We may add any function of the number of molecules to the entropy of a homogeneous gas without altering the value of the mixing entropy. Thus, there are infinitely many non-extensive expressions of the entropy function of a homogeneous gas that yield the same mixing entropy.

Consequently, the extensivity of entropy should be regarded as independent from the mixing rule. According to Gibbs and many other authors, this extensivity is nearly as obvious as the extensivity of energy because no sensible property is altered when two initially separated masses of the same gas at the same pressure and the same temperature are brought to communicate, for instance through an opening on the wall that separates them. Moreover, Gibbs’s theory of chemical equilibrium seems to require extensive entropies since it appeals to the entropy of mixtures of variable chemical composition. In this case, unlike the case of non-interacting mixtures, the way in which the entropy depends on the numbers of molecules is instrumental, and it seems plausible that non-extensive variants of the entropy function would derail the deduction of the equilibrium state.

These seemingly obvious arguments in favor of extensivity do not withstand criticism. In 1883, soon after Helmholtz published his own chemical thermodynamics, Boltzmann published a long memoir [53] in which he derived the laws of chemical equilibrium through his combinatorial method of 1877. In this approach, the basic entities are the atoms of which the various interacting molecules are made, and chemical equilibrium is obtained by maximizing the combinatorial probability of a combination of the atoms into molecules. Consider the simplest case of the dissociation equilibrium of a diatomic gas, say $I_{2}↔I+I$ for iodine. Call N′ the number of isolated atoms in the gas mixture, N″ the number of diatomic molecules, and *A* the total number of atoms (with N′+2N″=A). Since in Boltzmann’s combinatorics the atoms are mutually distinguishable, their permutation generally leads to a different combination for a given value of N′ and N″. The number *Y* of distinct combinations is the total number of permutations of the atoms divided by the number of permutations that leave the diatomic molecules invariant, by the number of permutations of the free atoms, and by the number of permutations of the diatomic molecules:

(47) $$Y=\frac{A!}{{\left(2!\right)}^{N\prime \prime }N\prime !N\prime \prime !}.$$

Each free atom is then distributed over the cells of its phase-space, and the same is done for the diatomic molecules. Call $N\prime_{i}$ the number of free atoms in the cell *i* of the first phase-space, and $N_{j}\prime \prime$ the number of molecules in the cell *j* for the second phase-space. For given values of the numbers N′ and N″ of free atoms and molecules, the number of distributions over their discretized phase-spaces are respectively given by

(48) $$W\prime =\frac{N\prime !}{\prod_{i}N_{i}\prime !}\;\mathrm{and}W\prime \prime =\frac{N\prime \prime !}{\prod_{j}N_{j}\prime \prime !}.$$

Boltzmann then gives the probability of a combination of the atoms compatible with these distributions as

(49) $$W\propto Y\;W\prime W\prime \prime =\frac{A!}{2^{N\prime \prime }}\frac{1}{\prod_{i}N_{i}\prime !}\frac{1}{\prod_{j}N_{j}\prime \prime !}.$$

The important point is that the factor *Y* cancels the N′! and N″! factors that are responsible for the non-extensivity of lnW′ and lnW″. Globally, Boltzmann’s combinatorial entropy lnW is not extensive since lnA! is not. Nonetheless, for a constant value of N′+2N″=A and for a constant value of the total energy, the maximal value of lnW depends on the degree of dissociation in the same manner as it would in Gibbs’s theory. Strangely (for the modern reader), it is precisely the distinguishability of the atoms in Boltzmann’s molecules that provides the desired N′! and N″! division (see [54]; [15], pp. 258–268).

The success of Boltzmann’s procedure incites us to reconsider the necessity of extensive entropies. Does communication between two similar masses of the same gas imply a global change of state? NO from a purely macroscopic point of view, and YES from a molecular point of view. Before the communication, the trajectory of a given molecule is confined to one half-space only; after the communication it penetrates both half-spaces. Now we have two options: either we want entropy to be a function of sensible states only, or we allow it to depend on the molecular microstates and their history. Gibbs overtly took the first option; Boltzmann’s relation between entropy and combinatorial probability seems to favor the second. The first one has mathematical appeal (it is more convenient to work with homogeneous functions) but it leads to the Gibbs paradox and it makes entropy depend on our present ability to detect the heterogeneity of a substance. The second annuls the Gibbs paradox since it yields exactly the same mixing entropy for identical gases and for different gases. But it seems to diminish the operational significance of entropy.

To summarize, the brute application of Boltzmann’s logarithmic relation between entropy and permutability leads to non-extensive entropies, and the reason for this is the distinguishability of molecules or atoms that is presupposed in the very definition of permutability. This is only a formal argument because the permutability can be divided by N! and the entropy is thus made extensive without changing its other properties. But there is more. Classical molecules are distinguishable in a more physical sense: any given molecule can be identified through its trajectory (that is, we can say whether two micro-objects, seen at different points at different times, correspond to the same molecule). In this context, the addition of two similar masses of gas is not a reversible operation and it is entirely similar to the mixing of two different gases. It would therefore seem that Boltzmann, for the sake of consistency, should have given up extensive entropies.

He did not. With the exception of the aforementioned communication to the *Philosophical magazine*, he always used extensive formulas for the entropy of a gas. In his view, this practice did not contradict the relation between entropy and probability because the permutability *W* was only meant to be proportional to the probability of the state, for a constant value of the numbers of ultimate particles. It could not be used to calculate the relative probability of states implying different numbers of ultimate particles. By ultimate particles, I mean the number *N* of molecules in the case of a homogeneous gas, and the list of the numbers *A*, *B*, *C*... of constituting atoms in the case of a mixture of chemically interacting molecules. The relation between entropy and probability should then be written as

(50) $$S=k_{B}\ln W+\varphi (N)\;\;\mathrm{and}\;\;S=k_{B}\ln W+\psi (A,B,C\ldots )$$

respectively. Extensive entropies obtain by setting the arbitrary functions ϕ and ψ to

(51) $$\varphi (N)=-k_{B}\ln N!\;\;\mathrm{and}\;\;\psi (A,B,C\ldots )=-k_{B}\ln(A!B!C!\ldots ).$$

As far as I know, no one defended non-extensive entropies until the new quantum theory reactivated the issue of the additive constant in the statistical entropy formula.

### 6.2. Absolute Entropies (1911–1920)

According to the combinatorial formula $S=k_{B}\ln W$, the value of the entropy also depends on the size of the cells into which the phase-space of a molecule is divided. Although this dependence is extensive, it is meaningless in a classical theory for which the size of the cells is largely arbitrary (according to Boltzmann, this size should be large enough to contain a large number of molecules and small enough so that the relative difference between the numbers of molecules in two contiguous cells be negligible). The quantum of action changed the game in the next century. In 1911, the Breslau physicist Otto Sackur [55] used Planck’s constant *h* to set the size of the cells and thus determined absolute entropies involving a *h*-dependent “chemical constant”. The hope was that in a chemical reaction involving gases only, the variation of the sum of absolute entropies would yield the true value of the entropy variation. This move supposed the elimination of any additive constant in the relation between entropy and probability. In particular, Sackur wanted his entropies to be extensive. Sackur achieved this aim by making the size of his quantum cells proportional to the number *N* of particles. The following year the seventeen-year old Dutch student Hugo Tetrode [56] derived the same entropy formula through the following adaptation of Gibbs’s microcanonical entropy:

(52) $$S=\frac{1}{N!}\frac{1}{h^{3N}}\int_{H<E}d^{3N}rd^{3N}p\;\mathrm{with}\;H=\sum_{i=1}^{N}\frac{p_{i}{}^{2}}{2m}$$

in the monatomic case. Tetrode justified the $h^{3N}$ divider through the quantum structure of phase space, and the N! division by appeal to Gibbs’s “generic phases,” about which more in a moment (see also [54], pp. 285–287).

Paul Ehrenfest, the guardian of Boltzmann’s heritage, protested that Sackur’s extensive cells or Tetrode’s N! division lacked any fundamental justification. In 1920 with his Czech assistant Viktor Trkal, he showed how to derive the laws of chemical equilibrium, including the full quantum theoretical expression of the equilibrium constant, without absolute extensive entropies [57] (see [58], pp. 51–55; [54], pp. 285–287). The basic idea was to return to Boltzmann’s method of 1883, the principal difference being in the size of the cells in the phase space of a molecule, now set to $h^{f}$ if *f* denotes the number of degrees of freedom of the molecule. Just as in Boltzmann’s original memoir, the division by the factorials of the molecule numbers is now justified by the multiplicity of ways in which distinguishable atoms can be combined to yield given numbers of molecules of each possible kind.

Ehrenfest seized this opportunity to publicize his criticism of entropy extensivity and entropy constants. About Sackur’s and Tetrode’s ways to achieve extensivity he wrote [57] (p. 163):

The law of dependence on N can only be satisfactorily settled by utilizing a process in which N changes reversibly and then comparing the ratios of the probability with the corresponding differences of entropy.

This process could be a chemical process occurring in a gas mixture, during which the numbers N′, N″,... of molecules of the various species can change reversibly. The equilibrium could then be computed in Boltzmann’s manner by computing the relative probability for the combination of atoms into the various species of molecules with abundance given by the numbers N′, N″,... :

Our method removes, we hope, any remaining obscurities as regards the occurrence of N′!N″!… This could only be accomplished, as it appeared to us, by not stopping at the numbers of the molecules in the combinatory computations, but by going down to the atoms.

Toward the end of the memoir, Ehrenfest condemned the usual justification of extensive entropies [57] (pp. 176–177):

In the majority of calculations of the chemical constants a special obscurity remains as to the way in which the ‘thermodynamic probability’ of a gas depends on the number of molecules. We shall try to explain in a few words how this obscurity is connected with the use of [Planck’s equation $S=k_{B}\ln W$ instead of Boltzmann’s $\Delta S=k_{B}\Delta \ln W$]: it is generally assumed as self-evident, that the entropy of a gas is to be taken twice as large, if the number of molecules and the volume are both doubled. Now it is certainly true, that the increase of the entropy in a given process in a gas of twice the number of molecules is twice as large as the corresponding increase in the original gas. But what is the meaning of taking the entropy itself twice as large and thereby settling the entropy-difference between the doubled and the original gas? By what reversible process is the double quantity of gas to be generated from the original quantity? Without that the entropy difference ∫δQ/T cannot be clearly defined. In order to remove this obscurity, it is necessary to return to Boltzmann’s equation [$\Delta S=k_{B}\Delta \ln W$] and to apply it to a reversible process in which the numbers of the molecules change.

Ehrenfest then reviewed the various procedures by Sackur, Tetrode, and judged them artificial. About Planck’s own justification of the N! division through the indistinguishability of the molecules he had to say: “I am not able—in spite of my sincere efforts—to grasp the foundation of the division by N! performed there” [59] (p. 628n).

Implicitly, Ehrenfest considered that the natural way of “doubling the quantity of gas”, by opening a hole on the wall between separating two equal portions of the gas, was not a reversible process. Logically, this should have led him to give a non-zero value to the mixing entropy of identical gases, and even to equate this value to the mixing entropy of different gases if he had the kinetic-molecular picture of the mixing in mind. He did not openly take this step, and he rather left the dependence of the entropy on the number of molecules undetermined for a single gas of stable composition.

Einstein agreed with Ehrenfest that the quantity of a gas could not be multiplied in a reversible process and that the usual demand of extensive entropies was “arbitrary”. In 1914 [60], he gave the entropy of a crystallized, chemically homogeneous substance at zero temperature as $k_{B}\ln(A!B!\ldots )$, wherein *A*, *B*,... denote the total number of atoms of each kind in the crystal. Implicitly, he thereby regarded $S=k_{B}\ln W$ as a valid tool for computing absolute entropies, and he regarded the atoms as distinguishable in the computation of *W*. Evidently, the resulting entropy is not extensive. He also noted that in the case of a mixture of two moles of two monatomic substances (for instance an alloy of two metals), the combinatorial entropy of the mixture differed from the combinatorial entropy of the separate substances by $k_{B}\ln[(2N_{A})!/N_{A}{!}^{2}]\approx 2R\ln2$. The result is of course the same if the two metals are identical. When transposed to gases at ordinary temperature, this reasoning gives non-extensive entropies and the same mixing-entropy for different and identical gases. Two years later, in a communication to the Physikalische Gesellschaft on the Sackur-Tetrode formula [61], Einstein felt free to introduce an N! division in order to get extensive entropies. But he did not relate this move to indistinguishability and he still regarded extensivity as an arbitrary (though convenient) demand.

### 6.3. Gibbsian Approaches (1902–1916)

Boltzmann’s and Ehrenfest’s approaches to chemical equilibrium implied the statistics of atoms and molecules when distributed over cells in the atomic or molecular phase space. In a more holistic approach, the gas or mixture of gases is considered as a whole mechanical system with a given Hamiltonian and the statistics refers to an ensemble of copies of the system. Somehow, averages over properly chosen ensembles are supposed to yield the desired thermodynamic properties. This is the essence of Gibbs’s method, which Boltzmann himself inaugurated but did not apply to chemical equilibrium. Gibbs’s *Statistical mechanics* of 1902 [62] was highly abstract and mathematical. It mostly concerned generic Hamiltonian systems with any given (large) number of degrees of freedom (*N*) and stationary ensembles built from them. Gibbs focused on two ensembles, the *microcanonical ensemble* distributed according to $\rho d^{N}qd^{N}p$, with

(53) ρ∝δ(E−H)

over the energy shell $H(q_{1},\ldots q_{N},p_{1},\ldots p_{N}=E)$ in the global phase space, and the *canonical ensemble* distributed according to $\rho d^{N}qd^{N}p$

(54) $$\rho \propto e^{-\beta H}.$$

He showed that averages based on these two stationary ensembles were mutually related in a manner analogous to thermodynamic quantities (see [63,64]).

In the fifteenth and last chapter of his treatise, Gibbs specialized the Hamiltonian to that of a set of molecules, with the intention to deal with the manner in which thermodynamic processes may involve molecule numbers. He had a special stake in this question since he had given the first thermodynamic theory of chemical equilibrium twenty-five years earlier. In analogy with the canonical ensemble in which the energy is distributed exponentially, he introduced the *grand-canonical ensemble* in which the molecule numbers can take different values with an exponential weight. Calling $\nu_{i}$ the number of molecules of the species *i*, $x_{i}$ their global phase, and $d\sigma_{i}$ the volume element of the associated phase space, the grand-canonical number of systems whose phase belongs to the element $\prod_{i}d\sigma_{i}$ of the global phase space is

(55) $$\begin{array}{c} \rho (\nu_{1},\ldots \nu_{s};x_{1},\ldots x_{s})\prod_{i}\frac{d\sigma_{i}}{\nu_{i}!}\;\;\mathrm{with}\;\;\rho =\Xi^{-1}e^{-\beta H(x_{1},\ldots x_{s})-\sum_{i}\mu_{i}\nu_{i}}\;\;\mathrm{and}\;\;\\ \Xi =\sum_{\nu_{1},\ldots \nu_{s}}\int \rho \prod_{i}\frac{d\sigma_{i}}{\nu_{i}!}. \end{array}$$

Gibbs here divides the measure $\prod_{i}d\sigma_{i}$ by the product of the factorials of the numbers of molecules. His first justification reads [62] (p. 187):

First of all, we must define precisely what is meant by statistical equilibrium of such an ensemble of systems. The essence of statistical equilibrium is the permanence of the number of systems which fall within any given limits with respect to phase. We have therefore to define how the term phase is to be understood in such cases. If two phases differ only in that certain entirely similar particles have changed places with one another, are they to be regarded as identical or different phases? If the particles are regarded as indistinguishable, it seems in accordance with the spirit of the statistical method to regard the phases as identical.

In Gibbs’s terminology, all “specific phases” that differ only by permutations of molecules of the same species correspond to the same “generic phase.” From an abstract mathematical point of view, there is nothing to favor generic phases over specific phases, since ensembles are purely mental constructs. It is all a matter of “practical convenience,” Gibbs tells us. More exactly, the choice of generic versus specific phases should be dictated by the quality of the thermodynamic analogy it permits.

As Gibbs has proven in an earlier chapter, the canonical ensemble of parameter β bears an analogy with a thermodynamic system of temperature $\beta^{-1}$. In particular, when two canonically distributed ensembles are brought into thermal contact (thanks to a small interaction Hamiltonian for the two corresponding mechanical systems), the joint ensemble is (approximately) stationary if and only if the β parameter has the same value for each of the combined ensembles. Similarly, in his last chapter Gibbs proves that when two grand-canonical ensembles are allowed to exchange molecules of a given species (thanks to a small semipermeable channel), the joint ensemble is (approximately) stationary if an only if the μ parameters of this species have the same value for each of the combined ensembles. Here the analogy is with the equality of the chemical potentials of a given chemical on the two sides of a semipermeable wall. It holds only if the generic concept of phase is adopted, as we might have expected from the intuitively evident fact that the joint ensemble is not stationary with respect to specific phases: a given (labeled) molecule is no longer confined to one of the two separate systems after they have been brought to communicate.

The expression that Gibbs gives for the grand-canonical entropy,

(56) $$S=-\sum_{\nu_{1},\ldots \nu_{s}}\int \rho \ln\rho \prod_{i}\frac{d\sigma_{i}}{\nu_{i}!}$$

is an extensive function of the grand-canonical averages ${\bar{\nu }}_{i}$ of the numbers $\nu_{i}$, as he wished it to be in analogy with the thermodynamic entropy used in his earlier theory of chemical equilibrium in gas reactions. Gibbs proves that this expression is very nearly equivalent to

(57) $$\begin{array}{c} S_{c}{}^{\mathrm{gen}}=-\int \rho_{c}{}^{\mathrm{gen}}\ln\rho_{c}{}^{\mathrm{gen}}\prod_{i}\frac{d\sigma_{i}}{{\bar{\nu }}_{i}!}\;\;\mathrm{with}\;\;\rho_{c}{}^{\mathrm{gen}}=Z_{\mathrm{gen}}{}^{-1}e^{-\beta H}\;\;\mathrm{and}\;\;\\ Z_{\mathrm{gen}}=\int e^{-\beta H}\prod_{i}\frac{d\sigma_{i}}{{\bar{\nu }}_{i}!}, \end{array}$$

which is the generic-phase version of the canonical entropy (the index c refers to the canonical ensemble, and “gen” refers to generic phases).

Gibbs ends his treatise with the situation of the Gibbs paradox [62] (pp. 206–207):

To fix our ideas, let us suppose that we have two identical fluid masses in contiguous chambers. The entropy of the whole is equal to the sum of the entropies of the parts, and double that of one part. Suppose a valve is now opened, making a communication between the chambers. We do not regard this as making any change in the entropy, although the masses of gas or liquid diffuse into one another, and although the same process of diffusion would increase the entropy, if the masses of fluid were different. It is evident, therefore, that it is equilibrium with respect to generic phases, and not with respect to specific, with which we have to do in the evaluation of entropy, and therefore, that we must use the average of [lnρ] or of [$\ln\rho_{c}{}^{\mathrm{gen}}$] and not that of [$\ln\rho_{c}$], as the equivalent of entropy, except in the thermodynamics of bodies in which the number of molecules of the various kinds is constant.

The remark echoes Gibbs’s earlier remark that entropy should refer to sensible properties only. Gibbs adjusts his definition of the statistical entropy so as to meet this requirement.

As was mentioned, in his justification of the N! division in quantum-theoretical context, Tetrode adduced the microcanonical version of Gibbs’s generic-phase entropy. In 1916, Planck [65] similarly relied on the generic-phase partition function (his *Zustandssumme*, from which the letter *Z* has survived) for a homogeneous gas:

(58) $$Z=\int e^{-\beta H}\frac{d\sigma }{N!},$$

in which dσ is the canonical measure in the phase space of the whole gas and *N* the number of molecules of the gas. The resulting free energy, $F=-\beta^{-1}\ln Z$, is found to be extensive. In a reply to Ehrenfest and Trkal, Planck [66] explained that statistical methods, when applied to the whole gas and not to its individual molecules, did not require the combinatorial distinguishability of the molecules and that the analyst should be free to choose the measure in phase-space that best fits his purpose. In his opinion, Ehrenfest was too narrow an empiricist when he required the extensivity of entropy to be justified by actual processes [66] (p. 367):

It does not help to wrack one’s brain on the meaning of a quantity for a process that does not exist in nature, but one may be satisfied with the following criterion: this quantity will be relevant if its calculated theoretical value for every process which can really be observed agrees with the measured value.

Mathematical physicists like Lothar Nordheim and David Enskog, or mathematicians like David Hilbert, favored the *N*! division. Other physicists believed the thermodynamic properties of a system should not be more determined than allowed by the underlying dynamical model, and they approved the standpoint of the Ehrenfest and Trkal paper. Albert Einstein did so in a letter to Ehrenfest, with a broader condemnation of Planck’s use of probabilities [67]:

Planck will not be talked out of his metaphysical probability concept. For whoever tries to understand minds of his kind, there is always a left-over irrationality, which escapes assimilation (which keeps reminding me of Fichte, Hegel, etc.).

By “metaphysical probability” Einstein meant: probability unjustified by proper physical argument, and perhaps justified by metaphysical arguments of the kind delivered by philosophers he did not like (see [54], pp. 288–290).

### 6.4. Foundations

Beyond rhetoric, Einstein was here pointing to a genuine difficulty of statistical mechanics in its various guises. In Boltzmann’s combinatorial approach of 1877 as well as in Gibbs’s statistical mechanics, the selected statistical distributions lack fundamental justification. Why should the mathematical permutability of a state truly measure its probability in a physical sense? Why should Gibbs’s stationary ensembles truly represent the equilibrium properties of a single system? How should the entropy be related to the probability distributions? Without proper answers to these fundamental questions, the users of statistical mechanics felt free to adjust the probability distributions in an ad hoc manner. In Einstein’s opinion, Planck had repeatedly indulged in this sort of opportunism (firstly in his derivation of the black-body law in 1900), and so too had Sackur, Tetrode, and their followers. In his own reflections on the foundations of statistical mechanics, published in 1902–1904, Einstein meant to remove any arbitrariness in the definition of the underlying probabilities and in their relation to entropy.

Boltzmann had already done so in writings overlooked by Einstein (see [4] (pp. 128–133, 245–248, 249–256). In 1881 Boltzmann [68] had shown that his combinatorial probabilities derived from the microcanonical distribution of the entire gas: the equiprobability of complexions is the discrete version of the uniformity of the microcanonical distribution over the energy shell. In addition, Boltzmann knew that for an ergodic system the temporal distribution of the phases of the system is microcanonical in the long run. Since he doubted that molecular systems were truly ergodic, in 1881 [69] he replaced ergodicity with the weaker assumption that within large but empirically accessible times the macroscopic properties of a system reaches a very nearly stable value independent of the choice of the initial conditions, as long as these conditions remain compatible with the macroscopic constraints. In other words, he assumed that the microdynamics was compatible with the empirically well-established existence of a well-defined, unique equilibrium state of a system under given macroscopic conditions. This assumption in itself implies that any stationary ensemble compatible with the macroscopic conditions should yield the macroscopic equilibrium properties of a system as averages over this ensemble. The microcanonical ensemble does the job under the constraint of constant total energy. The canonical ensemble does the job for a small subsystem of a system of the former kind. As Boltzmann knew since 1871 [70], an expression of the microcanonical and canonical entropies can be derived by considering slow deformations of the system in which the volume (and other parameters of the Hamiltonian) and the energy or temperature vary continuously. The entropy is thereby defined up to an additive constant depending on the (fixed) value of the numbers of molecules.

Let us try to apply these insights to the chemical equilibrium of a mixture of gases. Boltzmann’s method of 1883 is perfectly justified in a classical context, since it derives from the microcanonical distribution applied to all possible combinations of the constituting atoms. It does not spontaneously lead to an extensive entropy since it concerns only the state distribution for a given value of the total numbers of constituting atoms (whether one takes the logarithm of the combinatorial probability or the more direct and more fundamental formulas for the microcanonical or canonical entropy). But the entropy can be made extensive by subtracting from its expression the sum of the logarithms of the factorials of these numbers.

To summarize, in statistical mechanics at its most fundamental level, there seems to be nothing to tell us how the entropy should depend on the unchanging number of the ultimate particles (the molecules when there are no chemical reactions, and the atoms in general). In conformity with Boltzmann’s and Ehrenfest’s views, it is nevertheless possible to determine how the entropy depends on the composition of a mixture of chemically interacting substances. This dependence does not require extensive entropies. One may either favor non-extensive entropies in conformity with the molecular intuition, or extensive entropies in conformity with molar intuition. As we will see in Section 7, the choice between these two options is not only a matter of taste.

### 6.5. The Bose-Einstein Gas (1924–1925)

In the classical case, the molecular intuition led Ehrenfest and to tolerate non-extensive entropies. As Ehrenfest probably imagined and as his followers made clear, the molecules of a homogeneous gas can be distinguished through their path, so that the opening of a channel between two vessels alters the identity of the molecules that can be found in each vessel. Also, the combinatorial entropy formula $S=k_{B}\ln W$, as used in Boltzmann’s manner, assumes distinguishable molecules in a combinatorial sense and yields a non-extensive entropy. Ehrenfest preserved the intuition and the formula in the quantum theory because he reasoned in a semi-classical context in which molecules were still considered as classical objects with ad hoc quantization of their classical attributes.

In 1924, Einstein [71] offered a new theory of the quantum gas based on a combinatorial procedure from Satyendra Nath Bose’s earlier theory of black-body radiation as a gas of lightquanta (see [54], pp. 92–93). In this case, the formula $S=k_{B}\ln W$ directly yields an extensive entropy. As Ehrenfest and Einstein soon understood, Bose’s combinatorics presupposes indistinguishable particles for which Boltzmann’s permutability becomes unusable. A couple of years later quantum mechanics gave up the notion of a definite trajectory of the gas molecules, so that the intuitive molecular argument for the irreversibility of the mixing of two masses of the same gas lost its grab. The extensivity of entropy was no longer questioned, and the Gibbs paradox came back with increased vigor.

There even was a worse mixing paradox, introduced by Einstein at the end of his first memoir on the Bose-Einstein gas [71]. According to the new combinatorics, a mixture of two gases containing N′ particles of a first kind and N″ particles of a second kind essentially differs from a homogeneous gas of N′+N″ molecules in the same volume and at the same temperature. Not only the entropies but also the pressures differ because the low-temperature degeneracy does not occur at the same rate in the two compared gases. Einstein ended his memoir with the words (p. 267):

To conclude, I would like to bring the reader’s attention to a paradox which I have not been able to solve. There is no difficulty, with the method here given, to also treat the case of a mixture of two different gases. In this case, each kind of molecules has its own [quantum] ‘cells.’ Hence follows the additivity of the entropies of the components of the mixture. Thus, each component behaves as if it were alone [in the container] regarding molecular energy, pressure, and statistical distribution. A mixture with the molecule numbers N′ and N″, wherein the molecules of the first and second kinds differ as little as one wishes (in particular with respect to the molecular masses m′, m″) therefore gives, at given temperature, a pressure and a state-distribution different from those of a homogeneous gas with the molecule number N′+N″ and with practically the same molecular mass and the same volume. This seems virtually impossible [*so gut wie unmöglich*].

In his second memoir on the quantum gas [72] (p. 10), Einstein related gas degeneracy to the interference of de Broglie waves and argued that this interference would not occur for different molecules, even if the mass difference m′−m″ was very small, because the associated frequency difference $(m\prime -m\prime \prime )c^{2}/h$ could still be very large. In mature quantum mechanics, the argument is meaningless since the waves of different molecules should be multiplied, not added. So too is a later argument by Ehrenfest and George Uhlenbeck [73] based on an incorrect correspondence between the statistics of the molecules and their mutual impenetrability.

### 6.6. Von Neumann’s Solution

In his famous treatise of 1932 on the mathematical foundations of quantum mechanics [74] (pp. 191–202), Johann von Neumann claimed to have solved the Gibbs paradox (without naming it) by proving that the separability of the two components of a gas mixture, each represented by a different quantum state, depended on the distance between these states: separation was of course impossible for identical states, completely possible for orthogonal states, and partially possible for intermediate states. “We thus clarify an old paradox of the old form of thermodynamics, that is, the unpleasant discontinuity in the operation of semipermeable walls: states that differ as little as one wishes are nonetheless 100% separable, and exactly identical states are not at all separable!” (pp. 197–198). Von Neumann’s difficult argument may be summarized as follows.

Von Neumann considers a statistical ensemble in which each of the *N* (identical, typically macroscopic) systems of the ensemble is in a pure quantum state. Formally, the ensemble is represented by a density matrix of the form

(59) $$\rho =\frac{1}{N}\sum_{i=1}^{N}|\psi_{i}\rangle \langle \psi_{i}|\;\;(\mathrm{with}\;\mathrm{tr}\rho =1).$$

Von Neumann further imagines that each of the systems is placed in an ideal shell (say a potential barrier) so that is does not interact with the other systems (as should be the case in an ensemble), and he allows all the shells to move in a container. The motion is the thermal agitation implied by interactions of the shells with the thermalized walls of the container. The set of shells is thus analogous to a gas at a well-defined temperature. The thermal motion of this gas is completely decoupled from the evolution of the quantum states within the shells. When needed for the argument, the shells can be opened so that a measurement can be performed on the included system or an external field can be applied to it.

Firstly, von Neumann proves that the ensembles represented by any two pure states ρ′=|ψ′〉〈ψ′| and ρ″=|ψ″〉〈ψ″| have the same entropy. For this purpose, he sets the temperature of the gas of shells to zero and he imagines that all the systems of the first ensemble undergo a Hamiltonian evolution that turns |ψ′〉 into |ψ″〉, or, better in his mind, that all the systems are subjected to the same dense series of measurements with outcomes intermediate between ψ′ and ψ″. In the first case, the entropy does not change because the process is reversible and athermal, in the second case the process may be irreversible and the entropy in the end state may be higher than in the initial state, but it must nevertheless be the same because a similar process can be imagined from |ψ″〉 to |ψ′〉. Since the entropies of all pure states are equal, von Neumann takes their common value as a reference and thus sets the entropy of any pure state to zero.

Secondly, von Neumann considers the mixed ensemble of density

(60) ρ=a|φ〉〈φ|+b|ψ〉〈ψ|  with  a>0, b>0, a+b=1

and proves that the ϕ and ψ components can be separated by a semipermeable wall in the gas of shells as long as the orthogonality condition 〈ϕ|ψ〉=0 is met. For this purpose, he imagines a porous wall with holes slightly larger than the shells. The temperature of the gas of shells is now considered high enough for this gas to be ideal. Owing to their thermal motion, the shells occasionally penetrate a hole in the wall. Each hole is equipped with a mechanism that can measure the observable defined by

(61) O=|ϕ〉〈ϕ|−|ψ〉〈ψ|.

O-measurement of the states |ϕ〉 and |ψ〉 is non-destructive since 〈ϕ|ψ〉=0). When the outcome of the measurement is 1, the ball is sent to the other side of the wall with no change of momentum; when the outcome is −1 it is sent back with reflected momentum to the side it came from. According to von Neumann this operation can be done without entropy cost because the mechanism, unlike Maxwell’s demon, reacts on the instantaneous state of the shell in an automatic manner. Therefore, the wall behaves like the usual semipermeable walls with respect to the gas of shells, and it can be used for the purpose of reversible, isothermal separation of the ϕ and ψ gas components.

Thirdly, von Neumann proves that there cannot exist a semi-permeable wall for the separation of the ϕ- and ψ-gases if the corresponding states |ϕ〉 and |ψ〉 are not mutually orthogonal (The following is a much-simplified version of von Neumann’s lengthy reasoning). If such a wall existed, then by the usual thermodynamic reasoning the entropy $2Nk_{B}\ln2$ would be created by mixing *N* shells of the ϕ-gas with *N* shells of the ψ-gas. The entropy in the initial state is zero since the entropy of a pure state has been set to zero. As for the entropy in the final, mixed state $\rho_{f}=\frac{1}{2}|\phi \rangle \langle \phi |+\frac{1}{2}|\psi \rangle \langle \psi |$, it is given by

(62) $$S_{f}=-2Nk_{B}\mathrm{tr}(\rho_{f}\ln\rho_{f}).$$

For the derivation of this entropy formula, von Neumann sends the reader to the next part of his argument. The eigenvalues of $\rho_{f}$ being (1±α)/2 with α=|〈ϕ|ψ〉|, we have

(63) $$S_{f}=2Nk_{B}f(\alpha )\;\;\mathrm{with}\;\;f(\alpha )=-\frac{1+\alpha }{2}\ln\frac{1+\alpha }{2}-\frac{1-\alpha }{2}\ln\frac{1-\alpha }{2}.$$

The function f(α) decreases monotonously between f(0)=ln2 and f(1)=0. Therefore, the value of $S_{f}$ agrees with the mixing entropy $2Nk_{B}\ln2$ if and only if α=0. That is to say, complete separation is possible if and only the vectors |ϕ〉 and |ψ〉 are orthogonal.

Fourthly and lastly, von Neumann proves

(64) $$S=-Nk_{B}\mathrm{tr}(\rho \ln\rho )$$

for the entropy of a gas of *N* shells with the density matrix ρ. This matrix being Hermitian (and of trace 1), it may always be represented as a mixture

(65) $$\rho =\sum_{i}^{}p_{i}|i\rangle \langle i|\;\;\mathrm{with}\;\;\langle i|j\rangle =\delta_{ij}\;\;\mathrm{and}\;\;\sum_{i}p_{i}=1$$

of mutually orthogonal pure states. By von Neumann’s second result, there are walls that are permeable to one of the *i*-gases and impermeable to all the other *i*-gases. Using Planck’s double piston, we can separate the mixture of these gases isentropically, in such a manner that they all occupy in the end the same volume *V* as the original volume of the mixture. Spending the work $-Nk_{B}Tp_{i}\ln p_{i}$, we may then compress (isothermically) the *i*-gas to the fraction $p_{i}$ of the volume *V*. The entropy of this gas thereby varies by $Nk_{B}p_{i}\ln p_{i}$. By reasoning similar to the one given in the first step (except that the temperature of the gas now has a non-zero value), this entropy is unchanged if the |i〉 state is replaced by any given pure state, say |0〉. The same operations being performed on each of the *i* components, we end up with fractions of the same 0-gas at the same density and pressure. They may be joined together to form a gas of *N* shells in the |0〉 state. The entropy of such a gas having been set to zero, the entropy of the initial mixture must be

(66) $$S=-Nk_{B}\sum_{i}p_{i}\ln p_{i}=-Nk_{B}\mathrm{tr}(\rho \ln\rho ).$$

This result ends von Neumann thermodynamic considerations. The reasoning is ingenious but odd, because it purports to define a thermodynamic entropy for an ensemble that does not represent a state of equilibrium. Von Neumann’s density matrix is not constrained in any manner, and its Hamiltonian evolution is not considered. The shells in which the systems are enclosed do not have any degrees of freedom other than those of global translation, and there is no thermal coupling between the systems, the substance of the shells, and the thermostat with which the gas of shells is in contact. Consequently, it is not clear what von Neumann truly proved. His model is too far from a genuine gas model and his thought-experimentation is too unbridled to provide a convincing elucidation of the Gibbs paradox. Right after announcing such elucidation, von Neumann writes: “Here we should point out that the adventurous character of our ‘thought experiments,’ that is, the impossibility of their practical realization, does not hamper their demonstrative force: in the sense of phenomenological thermodynamics, every thinkable process has force of proof as long as it does not contradict the principles [the two laws of thermodynamics]” [74] (pp. 191–192). This may be wishful thinking if the thought experiments venture too far from any thinkable thermal process (see [75]). Yet we will see in a moment that von Neumann’s reasoning, if properly modified and reinterpreted, contains two keys to a quantum mechanical resolution of the Gibbs paradox.

## 7. Various Gibbs Paradoxes and Possible Solutions

### 7.1. In Macroscopic Thermodynamics

The history of the Gibbs paradox invites us to distinguish between three versions of the paradox belonging to three different levels of theory (Simon Saunders [74] Sections 1.2 and 1.3 has two levels, corresponding to my levels 2 and 3). The first level is the operational level based on an operational formulation of the two laws of thermodynamics and on the concepts of heat, work, and transformation. At this level, we have the paradox

**P_1_ :** *The maximal work that can be produced by natural mixing of two moles of different perfect gases at constant temperature without change of pressure has the same value *2RTln2*, no matter how small the difference between the two gases.*

This sounds like a paradox because no work can be produced by the natural mixing of two moles of the same gas and because we expect the case of identical gases to be the limit of vanishingly small difference between the two gases (continuity principle) (By continuity principle, I here mean the principle according to which a physical quantity varies continuously when the parameters of the system vary continuously. As pointed out by a referee, there are many examples of jumps of properties during a continuous variation of a system. For instance, a new symmetry appears when the sides of a rectangle become equal. However, in this case the jump is only qualitative. A measured quantity such as the surface of the rectangle varies continuously). The solution is easily found by considering the way in which the maximal work is computed. This computation requires the existence of a reversible means of mixing and separating the two gases, which itself presupposes the existence of an exploitable difference between the two gases. As long as no such difference is known, the two gases appear identical, we do not even think of separating them, and no work can be produced by their mixture. Thus, the possibility of producing work by isothermal mixing is contingent on our technical ability to detect heterogeneity. There truly is no paradox in P_1_ because a vanishingly small difference is undetectable and because the continuity principle obviously does not apply to contingently determined properties.

The second level of theory adds the entropy concept to the more primitive thermodynamic concepts. The corresponding version of the Gibbs paradox is

**P_2_** **:*** The thermodynamic value of the mixing entropy of two moles of different perfect gases is *2Rln2 *no matter how small the difference between the two gases.*

This will be a paradox if the calculation of the mixing entropy is justified and if entropy is an extensive quantity. The discussion is now more complex because there is no consensus on how entropy should be defined even at the level of macroscopic thermodynamics. It is commonly accepted that during a quasi-static, reversible transformation of a system exchanging the heat δQ with the source of temperature *T* at a given moment of the transformation, the entropy variation should be given as ∫δQ/T. But different authors disagree on how ideal the transformations that serve to determine the entropy function can be. They also disagree on what counts as a reversible transformation. In the present case, the calculation of the mixing entropy requires a real or ideal way of achieving reversible isothermal mixing. The real way appeals to condensation, chemical absorption, or gravity. The ideal way appeals to semipermeable walls. We could limit ourselves to the real way, and conclude, as we did for the maximal work gained by mixing, that the value of the mixing entropy hinges on the existence of concrete methods to detect and exploit heterogeneity. As Simon Saunders has said of this position [76] (p. 345), “the meaning of the entropy function does not extend beyond the competencies of the experimenter”. This is a first escape from paradox P_2_. The only problem is that this escape makes entropy a changeable property of a system, depending on our temporary ability to manipulate the system. Maxwell came close to this view in his encyclopedia article on diffusion (This is also the view defended by Edwin Jaynes [77]. A few authors [78,79] invoke the time needed for the interdiffusion and for the separation, arguing for instance that the time needed for separation by semipermeable walls grows indefinitely when the difference between the two gases goes to zero. Kenneth Denbigh and Michael Redhead [80] propound that an infinite number of steps are needed to separate two gases reversibly when the difference between the two gases goes to zero).

Alternatively, we may allow idealized entropy calculations and regard any conceivable difference between two gases, no matter how small, as exploitable for reversible separation, for instance through ideal semipermeable walls. This gives the mixing entropy a completely defined value for any imaginable pair of gases. Now, there are still three escapes from P_2_. We may, as Wiedeburg and Planck did, exclude the possibility of arbitrarily small differences between two gases. The variety of atoms and molecules is a discrete one in this view. In the second escape, proposed by Percy Bridgman [81] in 1941 (and anticipated by Wiedeburg), entropy is made to depend on “the universe of operations” through which it is ideally determined (Nowadays there are information-theoretic variants of this view. See [82] (pp. 28–29) for instance). Since this universe varies discontinuously in the limit of vanishing difference between the two gases, the discontinuity of the mixing entropy expressed in P_2_ is no longer a surprise.

The third escape, taken by Ehrenfest, is to deny the extensivity of (gas) entropy. For Gibbs and many others, the entropy should be extensive because entropy variations are extensive (since the exchanged heat is so) and because the entropy of a system should be a function of the sensible properties of the system only. In their opinion, there is no sensible difference in the state of a gas when it occupies two different chambers with the same pressure and temperature and when the two chambers communicate through an opening on the wall separating them. However, we have already denied that entropy depends only on sensible properties by allowing the mixing entropy to be defined by purely ideal transformations. So we may as well assume the existence of insensible processes occurring when the two chambers are brought to communicate and associate an entropy increase with these processes. Then we can adjust the mixing entropy of two moles of the same gas to the value 2Rln2 in order to avoid P_2_. Admittedly, the move is far-fetched as long as we do not have the kinetic-molecular picture in mind.

### 7.2. In Classical Statistical Mechanics

So far, we have considered macroscopic quantities only and we have refrained from any underlying mechanical model. The third level of analysis involves the kinetic molecular picture of a gas or its quantum-mechanical counterpart. We now have the paradox

**P_3_** **:** *The statistico-mechanical value of the mixing entropy of two moles of different perfect gases is* 2Rln2 *no matter how small the difference between the two gases.*

In this statement, the entropies are determined through one of the available statistico-mechanical methods. A priori, P_3_ should be distinguished from P_2_ because there need not be a perfect coincidence between the thermodynamic entropy and the statistical entropy. For statistical mechanics to yield a good approximation of thermodynamic laws, we only need a partial overlap of the two concepts. We know in advance that the overlap cannot be complete, since the statistical entropy cannot both concern a single closed system (as the thermodynamic entropy does) and be forever increasing in time. As Gibbs puts it, “the impossibility of an uncompensated decrease of entropy seems to be reduced to an improbability.”

Let us first consider the case of classical statistical mechanics, in which the gas system is represented by a set of molecules interacting according to the laws of classical mechanics. There are different approaches and different entropy formulas. Let us start with the elementary combinatorial approach of Boltzmann 1877, in which the relevant probability *W* is given by the number of ways of distributing *N* (distinguishable) molecules over cells in the phase space of a molecule and the associated entropy is given by $S=k_{B}\ln W$. As we are interested in the case of equilibrium only, we use the maximal value of *W* corresponding to the most probable distribution (Maxwell’s). This formula leads to a non-extensive entropy that can be made extensive by subtracting $k_{B}N\ln N$ from its value or, equivalently, by dividing *W* by N!. Boltzmann feels free to do so, plausibly because his main purpose it to derive Maxwell’s distribution as the most probable one and because the *N* dependence of the probability is here irrelevant (as we saw, Ehrenfest later argued similarly). The same argument, however, could be applied to the total energy *E* and yet the dependence S(E) is crucial in identifying $k_{B}\ln W_{\max}$ with the entropy.

In fact, the basic rules of entropy determination in the combinatorial approach remain arbitrary as long as we do not derive these rules from a more fundamental approach in which firstly the probabilities have a clear physical meaning, and secondly their relation to the entropy is physically justified. The first condition is satisfied by assuming, as Boltzmann and Einstein did, that for a closed system the microcanonical distribution ρ∝δ(E−H) correctly represents the long-term average behavior of a single system, with negligible exceptions. The second condition is satisfied by considering, as Boltzmann and Einstein also did, the slow deformations of the system (implying slow variations of the volume *V* and the energy *E*), and computing the Clausius integral ∫δQ/T with $\delta Q=\delta E-\bar{\delta \chi }$ and $T\propto \bar{K}$, wherein *K* represents the kinetic energy of the system and χ its potential energy (so that H=K+χ). In the usual units, this integral has the well-defined value

(67) $$\delta S=k_{B}\delta \ln\int_{H<E}d\sigma ,$$

in which dσ is the canonical measure in global phase space. For a homogeneous gas with a very high number of molecules, the phase integral in this formula can be replaced by a few approximately proportional expressions:

(68) $$\int_{H<E}d\sigma \propto \int_{}\delta (E-H)d\sigma \;\propto \int_{E-\Delta E<H<E}d\sigma \;\propto \sum_{{\left(N_{i}\right)}_{i}}W[{\left(N_{i}\right)}_{i}]\;\propto W_{\max}.$$

This is how Boltzmann justifies the success of the naive combinatorial approach.

When this reasoning is applied to a single homogeneous gas of stable molecules, the number of molecules does not vary and the differential relation (67) determines the entropy up to the integration constant, which is an arbitrary function of the number *N* of molecules. An extensive entropy may be obtained by taking

(69) $$S=k_{B}\ln\int_{H<E}d\sigma /N!$$

in conformity with Gibbs’s and Tetrode’s appeal to generic phases. In the case of a mixture of two non-interacting gases, the differential relation (67) determines the entropy up to an arbitrary function of the numbers N′ and N″ of the two kinds of molecules. There seems to be no rationale for comparing this entropy with the sums of the entropies of each gas considered by itself, because reversible mixing does not belong to the category of deformations originally assumed in the derivation of relation (67).

A first way to circumvent this difficulty is to rely on the probability of fluctuations of a certain kind for the phases of a microcanonically distributed system. First consider a fluctuation in which all the molecules are found within the partial volume υ of a container of volume *V*. The probability of this fluctuation is given by the ratio ${\left(\upsilon /V\right)}^{N}$, which is the portion of the energy shell in phase space for which the positions of every molecule is restricted to the volume υ. Formally, this is the ratio of the two values of the phase integral

(70) $$I=\int_{}\delta (E-H)d\sigma$$

when the positions of the molecule are confined to the volumes υ and *V* respectively. It also obtains intuitively by remarking that the probability of a given molecule to be in the fraction υ/V of the total volume is equal to this fraction. Now, the subset of microstates defined by the fluctuation is strictly identical with the set of microstates of a gas in a vessel of volume υ (with the same energy and the same number of molecules). It is therefore tempting to regard the difference

(71) $$k_{B}\ln I(\upsilon )-k_{B}\ln I(V)=Nk_{B}\ln(\upsilon /V)$$

as the entropy variation of a gas when the volume of its container goes from *V* to υ. In this case we are not learning anything new since we already know by independent reasoning (by slow deformation) that the function $k_{B}\ln I(V)$ properly represents the volume dependence of the entropy.

Let us in general assume the *fluctuation principle* according to which the probability of a subclass of phases of the system that agrees with the class of phases for a given macrostate of another system properly yields the entropy of the latter state (by taking the logarithm of this probability). As is well known, this principle did wonders in Einstein’s hands (More exactly, Einstein applied the reverse principle according to which the thermodynamic entropy can be used to compute the probability of fluctuations. This is how he arrived at the lightquantum and at the wave-corpuscle duality for black-body radiation and for the Bose-Einstein gas). We will first apply this principle to an equimolar mixture of two different gases in the volume 2V. The microcanonical probability of a fluctuation in which all the molecules of the first gas are confined in one half of the container and the molecules of the second gas are confined in the other half is given by ${\left(1/2\right)}^{N}{\left(1/2\right)}^{N}$. The fluctuating phases being the same as the phases of a system made of two separate vessels for each gas in equilibrium (with the same energy or temperature), by the fluctuation principle the entropy of the mixture differs from the entropy of the separated gases by $2Nk_{B}\ln2$, in conformity with the classical value of the mixing entropy.

Now consider a single homogeneous gas in the volume 2V. The molecules being classically distinguishable by their trajectories, we may imagine a fluctuation in which the *N* first molecules of the gas are confined within one half of this volume and the *N* last molecules of the gas are confined within the other half. The probability of such a fluctuation is again ${\left(1/2\right)}^{N}{\left(1/2\right)}^{N}$ and its phases agree with the equilibrium state of a system made of two separate vessels each containing *N* molecules of the gas. By the fluctuation principle we therefore have

(72) $$S(2N,2V,T)=2S(N,V,T)+2Nk_{B}\ln2.$$

This entropy is not extensive, and the departure from extensivity is just what we need to avoid P_3_. This is a solution to the Gibbs paradox.

As was already mentioned, the Boltzmann-Einstein way to define entropy variations in statistical mechanics is to consider slow deformations of a system involving change of volume (and other parameters of the Hamiltonian), change of the energy (in the microcanonical case), and change of the β parameter (in the canonical case). The change of volume is effected by including in the potential χ of the forces acting on the molecules a potential barrier $\chi_{\lambda }(r)$ that mimics a wall (this function vanishes within the volume of the vessel and takes a very large value outside). The slow variation of the parameter λ shifts the barrier to produce the desired volume change. Now, within a vessel of constant volume we may imagine a potential barrier that blocks the molecules of one gas on one side of the barrier but does not act on the molecules of another gas. The potential barrier plays the role of a semipermeable wall and it can be used to derive an extension of the differential relation (67) to reversible mixing. Thus, the mixing entropy can be derived in statistical mechanics without recourse to the fluctuation principle. But we cannot imagine any semipermeable potential barrier in the case of identical gases (Dennis Dieks [83] (pp. 370–371) conceives semipermeable walls even in the case of two identical gases in the classical case. Such walls require Maxwell demons that defy concrete realization. Their mechanism should be made entirely explicit in order to precisely judge their statistico-mechanical effects. This is why I prefer to restrict my analysis to walls that can be modelized as potential barriers). The fluctuation principle therefore seems necessary to determine the effect of a doubling of the mass of a gas on its entropy, unless we can imagine a reversible way to alter the number of molecules.

Note in passing that the microcanonical entropy formula

(73) $$S=k_{B}\ln\int_{H<E}d\sigma$$

and derived formulas all give the departure $k_{B}N\ln N$ from extensivity in conformity with the fluctuation principle. This was to be expected since one of the derived formulas agrees with Boltzmann’s combinatorial entropy of 1877 and since the implied permutability and the application of the fluctuation principle both appeal to distinguishable molecules. Yet there does not seem to be any reason to take the $k_{B}N\ln N$ term seriously besides the fluctuation principle.

So far we have reasoned with the microcanonical distribution and the associated entropy formula. Another entropy formula can be obtained by regarding the system of interest as a small subsystem of a larger system described by the microcanonical distribution, the complementary system here playing the role of a thermostat. As was already known to Boltzmann, the small subsystem is distributed according to the canonical law

(74) $$\rho =Z^{-1}e^{-\beta H},\;\mathrm{with}\;Z=\int e^{-\beta H}d\sigma .$$

Considering small deformations of the latter system in which the volume V and the inverse temperature β now are variable parameters and computing the ratio

(75) $$\frac{\delta Q}{T}=k_{B}\beta \int (\delta \bar{H}-\bar{\delta \chi })d\sigma ,$$

we get

(76) $$\delta S=-k_{B}\delta \int \rho \ln\rho d\sigma .$$

Again, this formula determines the entropy up to any function of the numbers of molecules. We may again invoke the fluctuation principle and get the same results for the mixing entropy and the *N* dependence of the entropy of a homogeneous gas as we did in the microcanonical case.

Strictly speaking it is impossible to reversibly (quasi-statistically and continuously) alter a discrete quantity. However, we may consider systems in which the number of particles fluctuates around a well-defined average. This will be the case if the vessel of volume *V* containing the (homogeneous) gas communicates through a small channel with a much larger container. Call αV this volume and *M* the total number of molecules (with α>>1 and M>>1). We suppose the global system to be in equilibrium with a thermostat. The phases of the global system being distributed canonically, the probability that the *N* first molecules of the global system be in the volume *V* and in a phase belonging to the element $d\sigma_{N}$ of the phase space of *N* molecules is very nearly proportional to $e^{\beta \mu N}e^{-\beta H_{N}}d\sigma_{N}$ in which μ is a constant of the large container (just as the parameter β is a constant of the thermostat in the derivation of the canonical law). Now, if instead of the *N* first molecules we take any *N* distinct molecules picked among the *M* molecule in any order and require their phase to belong to $d\sigma_{N}$, we are still getting the same microstate of the gas contained in the vessel of volume *V* since the identity of the molecules contained in this vessel constantly changes and cannot be taken into consideration in the long run. Consequently, the probability that this system contains *N* molecules in a phase belonging to $d\sigma_{N}$ is proportional to

(77) $$e^{-\beta (H_{N}-\mu N)}\frac{M!}{N!(M-N)!}d\sigma_{N}\approx e^{-\beta (H_{N}-\mu N)}\frac{d\sigma_{N}}{N!}.$$

After normalization, this probability is equal to $\rho_{N}d\sigma_{N}/N!$, with

(78) $$\rho_{N}=\Xi^{-1}e^{-\beta (H_{N}-\mu N)}\;\mathrm{and}\;\Xi =\sum_{N=0}^{+\infty }\int e^{-\beta (H_{N}-\mu N)}\frac{d\sigma_{N}}{N!}.$$

The N! division here receives a similar justification as in Boltzmann’s reasoning for a chemical mixture (Nicolaas van Kampen gave a similar argument in 1984 [1]. See also [76], p. 351). As was mentioned, Gibbs introduced it axiomatically in the last chapter of his *statistical mechanics* to define the grand-canonical ensemble.

The consideration of deformations of the system in which the parameters V, β, and μ vary slowly leads to the entropy formula

(79) $$S=-k_{B}\bar{\ln\rho_{N}}=-k_{B}\sum_{N=0}^{+\infty }\int \rho_{N}\ln\rho_{N}\frac{d\sigma_{N}}{N!}=k_{B}\beta ({\bar{H}}_{N}-\mu \bar{N}-\Omega )\;\mathrm{with}\;\Omega =-\beta^{-1}\ln\Xi .$$

The average energy ${\bar{H}}_{N}$, the average molecule number $\bar{N}$, and the potential Ω are easily seen to be proportional to the volume *V*, so that the entropy is extensive (The fluctuations of the particle number and of the energy being negligible, the entropy is also given by the generic-phase versions of the canonical and microcanonical entropy). This should not surprise us since in the present approach the (average) particle number can be altered reversibly by slow variation of the μ parameter of the container. The reasoning can be generalized to a gas mixture that communicates through semipermeable walls with reservoirs containing the various components of the mixture. The result is Gibbs’s grand-canonical ensemble in its most general form.

We seem to be arriving at a contradiction: whereas in the microcanonical and canonical approaches supplemented with the fluctuation principle we got non-extensive entropies, in the grand-canonical approach we get extensive entropies. In reality, we have to do with two different physical situations. In the former case, the two masses of the same gas that are brought to communicate are originally in two sealed vessels. In the latter case, the two masses can be imagined to be originally communicating with a reservoir that determines the common value of their μ parameter so that the addition of a new, direct channel of communication does not bring any qualitative change. In other words, the molecules in the grand-canonical case are effectively indistinguishable since their trajectory does not permanently reside in the vessel. It makes no sense to say that a molecule that was originally confined in one vessel becomes able to visit the two vessels when an opening is made on the separating wall.

Does the extensivity of the grand-canonical entropy imply a resurgence of paradox P_3_? Not really, because what we have in mind in any version of the Gibbs paradox is the mixing of gases originally contained in separate sealed vessels. P_3_ makes sense only in microcanonical or canonical context. Then we have two ways to solve it (without invoking an essential discontinuity of chemical differences): either we accept the fluctuation principle and we agree that the natural mixing of two masses of the same gas creates just as much entropy as the natural mixing of different gases (as Dennis Dieks [83] does), or we decide (as Nicolaas van Kampen [1] does) that the statistical entropy is not sufficiently defined to decide whether entropy is extensive or not (As was mentioned in an earlier footnote, Dieks relies on a Maxwell demon, not on the fluctuation principle. Harold Grad adopts an intermediate position, in which the choices between the two options depends on a free choice of the level of information we include in the definition of the entropy [84], pp. 326–327. See also [85]).

### 7.3. In Quantum Statistical Mechanics

In quantum statistical mechanics, the remarks we earlier made in the classical case about the physical definition of probabilities and entropy variations still apply. However, the state of the gas is now defined by a density matrix instead of a point in phase space, and the micromodel of the gas is radically different. The absence of well-defined trajectories in this model, the perfect indistinguishability of the molecules, and the holistic character of the definition of the states seem to make it impossible to ascribe any meaning to the statement that a given molecule is in one half of the container and not in the other. Communication between two chambers containing the same gas at the same temperature and pressure still implies a change of state because the number of molecules in a given chamber was fixed before communication and fluctuates after communication (and also because the proper cavity modes are different before and after communication). However, the number of molecules being very large, this fluctuation is very small and we intuitively anticipate a negligible effective difference between the separate and fused states (see [86]).

Again, me may use the fluctuation principle to compute the associated entropy variation. This could be done for the canonical entropy by quantum-field-theoretical means. We will here reason more naively by comparing the number F(N,P) of distributions of *N* indistinguishable particles over *P* cells of size ε in the volume *V* with the number F(2N,2P) of distributions of 2*N* indistinguishable particles over 2*P* cells of size ε in the volume 2*V*. In the Stirling approximation we have

(80) $$F(N,P)=\frac{(N+P-1)!}{(N)!(P-1)!}\sim \sqrt{\frac{P}{2\pi N(N+P)}}\frac{{\left(N+P\right)}^{N+P}}{N^{N}P^{P}},$$

which gives

(81) $$\frac{F(2N,2P)}{F{\left(N,P\right)}^{2}}=\sqrt{\frac{N(N+P)}{\pi P}}.$$

The logarithm of this expression gives an entropy difference of the order of lnN, which is negligible compared to entropies of the order of *N* for the separate and fused states.

In agreement with this approximate lack of a mixing entropy, the canonical entropy is very nearly extensive. In quantum statistical mechanics, the canonical state is given by the density matrix

(82) $$\rho =Z_{}^{-1}\sum_{\sum_{r}n_{r}=N}e^{-\beta \sum_{r}n_{r}\varepsilon_{r}}|{\left(n_{r}\right)}_{r}\rangle \langle {\left(n_{r}\right)}_{r}|\;,\mathrm{with}\;Z_{}^{}=\sum_{\sum_{r}n_{r}=N}e^{-\beta \sum_{r}n_{r}\varepsilon_{r}},$$

in which the index *r* labels the proper wave modes of the cavity of volume V, $\varepsilon_{r}$ denotes the energy of the *r*-mode, and the integer $n_{r}$ denotes its excitation number (interpreted as the number of indistinguishable molecules attached to this mode). The big sum bears over every choice of the sequence ${\left(n_{r}\right)}_{r}$ of these numbers such that their sum equals *N.* The state obtained from the former by doubling *N* and *V* clearly differs from the product state for two separate vessels of volume *V* each containing *N* molecules. In symbols: $\rho_{2V,2N}\neq \rho_{V,N}⊗\rho_{V,N}$. The entropy $-k_{B}\mathrm{tr}\rho \ln\rho$ is nevertheless approximately extensive because the grand-canonical entropy is extensive and because this entropy is approximately equal to the canonical entropy. This may be seen as follows.

The grand-canonical partition function reads

(83) $$\Xi =\sum_{{\left(n_{r}\right)}_{r}}e^{-\beta \sum_{r}n_{r}(\varepsilon_{r}-\mu )}=\prod_{r}\sum_{n_{r}=0}^{+\infty }e^{-\beta n_{r}(\varepsilon_{r}-\mu )}=\prod_{r}{\left(1-e^{-\beta (\varepsilon_{r}-\mu )}\right)}^{-1}.$$

For the grand-canonical potential, this gives

(84) $$\Omega =-\beta^{-1}\ln\Xi =\beta^{-1}\sum_{r}\ln(1-e^{-\beta (\varepsilon_{r}-\mu )})$$

The density of modes being proportional to the volume *V*, we have

(85) Ω(αV,β,μ)=αΩ(V,β,μ) for any α,

that is, Ω is extensive. So too is the grand-canonical entropy $S_{\mathrm{gc}}=k_{B}\beta^{2}\partial \Omega /\partial \beta$. The same property holds for the average number of molecules $\bar{N}=-\partial \Omega /\partial \mu$. The fluctuations of the number *N* being very small, we may write

(86) $$\Xi =\sum_{N}e^{\beta \mu N}Z_{N}\approx e^{\beta \mu \bar{N}}Z_{\bar{N}}$$

in a sufficient logarithmic approximation. Using $F_{N}=-\beta^{-1}\ln Z_{N}$, this gives

(87) $$\begin{array}{c} F_{\bar{N}}\approx \Omega +\mu \bar{N}\;\;\;\mathrm{and}\\ F_{\alpha \bar{N}}(\alpha V,\beta )\approx \Omega (\alpha V,\beta ,\mu )+\mu \bar{N}(\alpha V,\beta ,\mu )\approx \alpha F_{\bar{N}}(V,\beta ). \end{array}$$

In this approximation the free energy is extensive and so is therefore the canonical entropy.

As for the mixing rule, we still have two possible justifications: through the slow shift of a semipermeable barrier, and through the fluctuation principle. The main difference is that we can no longer rely on the corpuscular picture when computing the probability of a fluctuation in which the two gases are entirely confined to their original volumes $V_{1}$ and $V_{2}$ (before mixing). This probability could still be computed by quantum-field-theoretic methods. For our purpose, it is sufficient to remark that by the fluctuation principle and for non-interacting, dynamically independent gases, this probability determines both the mixing entropy and the entropy created during the free expansion of the two gases from their original volumes $V_{1}$ and $V_{2}$ to their final volume $V_{1}+V_{2}$ (Of course, the value of the mixing entropy will depart from its classical value for low temperatures at which degeneracy occurs, and it vanishes at zero temperature since the entropy of a quantum gas does).

Paradox P_3_ therefore holds in full force in the quantum case. In addition, we get Einstein’s mixing paradox according to which the equation of state of a quantum gas (at low temperature) is altered in a discontinuous manner when we consider a mixture of two different gases instead of a single homogeneous gas with the same total number of particles. In this new paradox, all relevant properties are directly observable and we no longer have to fear the definitional traps of the entropy concept. Wiedeburg’s old suggestion that we should regard the chemical identity of a substance as an inherently discrete variable seems to be the only escape from Gibbs’s and Einstein’s paradoxes. From a quantum-theoretical point of view, different species correspond to different eigenstates of the same Hermitian operator, which implies the desired discreteness. They also obey superselection rules that yield the dynamical independence required for the computation of the mixing entropy.

However, instead of two different chemical species for the molecules, we may also consider two different internal states. Take for instance two gases of silver atoms with all the spins pointing in one direction for the first gas, and all the spins pointing in another (fixed) direction for the other gas. Since the direction of a spin eigenstate is a continuous variable, the difference between these two gases can be made arbitrarily small and we again face the Gibbs paradox. Alfred Landé [87,88], who first described this version of the paradox, also remarked that in this case the usual basis for deriving the mixing entropy is lacking: no complete separation by semipermeable walls is conceivable unless the two spin states are orthogonal, because a wall permeable to one spin state will let the other spin state through with a probability given by the squared modulus of their scalar product. Landé believed he could still compute the entropy of the mixture by analyzing it into two gases of opposite spin directions. For instance a mixture of $|{+}_{u}\rangle$ and $|{+}_{v}\rangle$ spin states in the directions u and v can be analyzed into a mixture of $|{+}_{u}\rangle$ and $|{-}_{u}\rangle$ spin states through “filters” that project the spin state of an incoming molecule onto the latter pair of spin states and let one of these states through while they reflect the other. As Dennis Dieks and Vincent van Dijk once remarked [75], this does not work because the action of these filters is irreversible: they turn a pure spin state into a mixture.

Yet the mixing entropy can still be defined and computed by transposing two of von Neumann’s ideas to the present system (The following reasoning is based on semi-permeable walls. Alternatively, it is possible to exploit a quantum-mechanical definition of the maximal work produced by mixing. This is the way in which Armen Allahverdyan and Theo Nieuwenhuizen [89] conceived their ingenious solution to the quantum Gibbs paradox). The first idea is that a mixture of two gases whose global spin states |ϕ〉 and |ψ〉 are factorized and orthogonal behaves like a mixture of two chemically different gases. This mixture formally allows reversible separation through walls represented by the potentials barriers of Hamiltonian

(88) $$H_{\varphi }=|\varphi \rangle \langle \varphi |\;\sum_{i=1}^{N}\chi (r_{i})\;\;\mathrm{and}\;\;H_{\psi }=|\psi \rangle \langle \psi |\;\sum_{i=1}^{N}\chi (r_{i}),$$

wherein $r_{i}$ is the position of the center of mass of the *i*th molecule and χ is a function that vanishes on one side of the barrier and takes a very large value on the other side. The second idea is that a mixture of two gases whose global spin states |ϕ〉 and |ψ〉 are factorized but non-orthogonal is equivalent to a mixture of two fully and reversibly separable gases in the proportion (1+α)/(1−α) with α=|〈ϕ|ψ〉|.

Take two chemically homogeneous gases of *N* molecules originally in separate, contiguous chambers of volume *V*. The molecules of the first gas are assumed to be all in the same spin state |θ〉 and those of the second gas in the same spin state |−θ〉, with

(89) |±θ〉=cosθ|+〉±sinθ|−〉.

If |+〉 and |−〉 denote the two spin eigenstates in a fixed direction of space (2θ is the angle that the spin axes of the states |θ〉 and |−θ〉 make with this direction). The spin states |θ〉 and |−θ〉 agree for θ=0 and their scalar product 〈θ|−θ〉=cos2θ decreases when θ grows from 0 to the value π/4 for which it vanishes. We are thus equipped to consider a continuous variation of the difference of the two gases between perfect identity and perfect separability.

Now let the two gases be mixed though an opening on the separating wall. If the ±θ-gas was alone in the thermostated vessel of volume 2V, its density matrix would be

(90) $$\rho_{\pm }=Z^{-1}\sum_{r}e^{-\beta E_{r}}|r\rangle \langle r|⊗{|\pm \theta \rangle }^{⊗N}{\langle \pm \theta |}^{⊗N}\;\mathrm{with}\;Z=N^{-1}\sum_{r}e^{-\beta E_{r}}\mathrm{so}\;\mathrm{that}\;\mathrm{tr}\rho_{\pm }=N.$$

Here the vectors |r〉 refer to any basis of stationary states of a spinless gas of *N* molecules in the cavity of volume 2V and $E_{r}$ denote the energy in the state |r〉. The symbol ${|\pm \theta \rangle }^{⊗N}$ stands for the spin state |±θ〉⊗|±θ〉…⊗|±θ〉 of the *N* molecules (symmetry here allows global factorization). Provided that the spins of the molecules of the two gases are not affected during collisions, the final state of the mixture will be

(91) $$\rho =\rho_{+}+\rho_{-}=Z^{-1}\sum_{r}e^{-\beta E_{r}}|r\rangle \langle r|⊗\left(\;{|\theta \rangle }^{⊗N}{\langle \theta |}^{⊗N}+{|-\theta \rangle }^{⊗N}{\langle -\theta |}^{⊗N}\right).$$

We now introduce the two orthogonal unit vectors

(92) $${|\pm \rangle }_{N}=\frac{{|\theta \rangle }^{⊗N}\pm {|-\theta \rangle }^{⊗N}}{\left|{|\theta \rangle }^{⊗N}\pm {|-\theta \rangle }^{⊗N}\right|}$$

as well as the parameter

(93) $$\alpha ={\langle \theta |}^{⊗N}{|-\theta \rangle }^{⊗N}={\langle \theta |-\theta \rangle }^{N}.$$

In these terms, we have

(94) $${|\theta \rangle }^{⊗N}{\langle \theta |}^{⊗N}+{|-\theta \rangle }^{⊗N}{\langle -\theta |}^{⊗N}=(1+\alpha ){|+\rangle }_{N}{\langle +|}_{N}+(1-\alpha ){|-\rangle }_{N}{\langle -|}_{N}.$$

Our mixture is therefore equivalent to a mixture of two gases whose molecules are globally in the orthogonal spin states ${|+\rangle }_{N}$ and ${|-\rangle }_{N}$. Reversible mixing of these two states being possible by a semipermeable wall, the corresponding mixing entropy is equal to the entropy created by the reversible expansion of a gas of N(1+α) molecules from the volume V(1+α) to the volume 2V plus the entropy created by the reversible expansion of a gas of N(1−α) molecules from the volume V(1−α) to the volume 2V. In the ideal, moderate-temperature case for which the Boyle-Mariotte law applies, this gives

(95) $$S_{m}=k_{B}N(1+\alpha )\ln\frac{2}{1+\alpha }+k_{B}N(1-\alpha )\ln\frac{2}{1-\alpha }.$$

When α grows from 0 to 1, this mixing entropy decreases from $2k_{B}\ln2$ to 0. The initial state in this mixing process has the same entropy as a homogeneous gas of 2*N* identically polarized molecules because entropy is extensive, additive, and does not depend on the state of polarization. So is too the entropy of the initial state for the mixing of θ and −θ molecules (It might be objected that for very large *N* the parameter $\alpha ={\langle \theta |-\theta \rangle }^{N}$ can have an extremely small value even when 〈θ|−θ〉 is still close to 1. This difficulty is removed by noting that in such cases the difference between the two gases should be measured by N(1−〈θ|−θ〉) instead of 1−〈θ|−θ〉). Consequently, the mixing entropy of the two mixing processes is the same and it is given by the former equation. The mixing entropy of two fully polarized gases therefore goes to zero when the difference of polarization goes to zero. The Gibbs paradox is solved. The Einstein mixing paradox is also solved in a similar manner, by noting that the mixture of two polarized gases is equivalent to a mixture of two fully separable gases and that the proportion of this mixture continuously approaches 1/0 when the difference between the polarizations goes to zero.

Lastly, a few words should be said on Erwin Schrödinger’s influential and yet confusing discussion of the Gibbs paradox in his Dublin lectures of 1944 [90]. There Schrödinger observed that classical statistically mechanics naturally led to the same mixing entropy for different and identical gases, and commented (p. 61):

It was a famous paradox pointed out for the first time by W. Gibbs, that the same increase of entropy [for the mixing of two different gases] must not be taken into account, when the two molecules are of the same gas, although (according to naïve gas-theoretical views) diffusion takes place then too, but unnoticeably to us, because all the particles are alike. The modern view solves this paradox by declaring that in the second case there is no real diffusion, because exchange between like particles is not a real event—if it were, we should have to take account of it statistically. It has always been believed that Gibbs’s paradox embodied profound thought. That it was intimately linked up with something so important and entirely new could hardly be foreseen.

What Schrödinger here calls “Gibbs’s paradox” is not the original paradox. It is the statement that molecular intuition and classical statistical mechanics lead to the same mixing entropy for two identical gases as for two different gases, at odds with the extensivity of entropy in macroscopic thermodynamics. This statement does not at all involve the limiting case of a vanishing small difference, and boils down to a comparison between the predictions of two different theories (macroscopic thermodynamics and classical statistical mechanics) for the relation between the entropies S(2V,2N,T) and S(V,N,T) of a homogeneous gas. We are quite far from Gibbs’s statement. Moreover, it is not true that Schrödinger’s paradox cannot be solved in classical context. It can be solved by giving up extensivity, as we have seen (According to Simon Saunders [76],pp. 352–353, it can also be solved by counting the distributions of indistinguishable molecules over elementary cells for a given distribution over macro-cells that contain many elementary cells. This is tantamount to deriving the extensivity of classical entropies from the extensivity of quantum entropies by taking the classical limit of the latter (the elementary cells here playing the role of quantum cells)). It is also untrue that in quantum context nothing happens when two samples of the same gas are allowed to communicate. As we saw, the communication increases the number of microstates accessible to the system, although this increase does not significantly alter the value of the entropy. Pace Schrödinger, the Gibbs paradox did not foreshadow quantum indistinguishability.

Was Schrödinger nonetheless right to oppose the indistinguishability of quantum particles with the distinguishability of classical particles? There is no consensus on this issue. It has often been remarked that indistinguishability can be defined and used in classical context (Gibbs in fact invented the word), and that conversely distinguishability can be introduced in quantum context [91,92]. In classical context, the instantaneous phase of a set of labeled point-like molecules is evidently invariant by permutation of the labels. It is even possible to describe the state of this set in a completely symmetrical manner, with no labeling at all, and to write the equations of evolution of the system for such intrinsic states [54] (p. 241n). In this sense, the molecules are indistinguishable. However, for derived thermodynamic quantities including the entropy, it cannot make any difference whether the molecules are labeled or not. The reason is that thermodynamic quantities depend on the long-term behavior of macro-properties that do not depend on the labeling. For instance, the distribution ${\left(N_{i}\right)}_{i}$ of the *N* the molecules over cells in molecular phase space does not depend on the labeling by definition, and its temporal probability, as derived from the microcanonical distribution of global phases, is the same whether these phases are defined specifically (with fixed labeling) or generically (in a permutation-invariant manner). Indeed the only effect of adopting generic phases instead of specific phases is to divide the canonical measure dσ in phase space by N!. Whether or not the molecules are labeled, the temporal probability of the distribution ${\left(N_{i}\right)}_{i}$ is proportional to Boltzmann’s permutability $N!/\prod_{i}N_{i}!$, whose naive interpretation relies on counting the distribution of labeled molecules over cells (hence Boltzmann’s word “permutability”). The reason for this paradoxical state of affairs is that classical molecules, even though they may be indistinguishable in the above-given sense, still are *traceable*: the long-term evolution of the global system, on which thermodynamic behavior crucially depends, implies distinct trajectories for each molecule. In classical statistical mechanics, distinguishability boils down to traceability.

In contrast, it has often been argued that the indistinguishability of quantum particles excludes traceability. It is indeed a commonplace of quantum theory that it excludes a well-defined trajectory of a particle in ordinary space. Against this, Simon Saunders and Dennis Dieks have argued [76,91,92], there are resources in quantum mechanics that allow us, at least in quasi-classical cases, to differentiate trajectories for the various molecules of a gas. Thus, the fully (anti)symmetric states that are usually meant to define quantum indistinguishability do not necessarily exclude traceability. The crucial point, however, is that the stationary states used in quantum statistical mechanics, for instance the canonical density matrix, do not allow such differentiation: the relevant quantized wave modes are completely delocalized in the volume of the cavity (This statement needs to be nuanced: in the case of a quasi-classical gas for which the de Broglie lengths of the molecules are much smaller than their mutual distances, the quantum state of the gas may be approximated by a state in which molecular trajectories are definable). Just as in the classical case, what truly matters is the long-term evolution of the global micro-model, and not the ad hoc combinatorial procedures that Boltzmann and Einstein used in their first statistico-mechanical derivations of the entropy of a gas.

## 8. The Relation between Theory and Experiments in the Light of the Gibbs Paradox

Discussions of the Gibbs paradox typically involve a fanciful mix of abstract theoretical notions, imagined devices and processes, and real experiments. Most of the confusion in the literature on this paradox comes from an insufficient understanding of the articulation of these three elements in physical theory in general. As I have argued elsewhere [93,94] (Chap. 9), a physical theory should in general be defined as a symbolic universe equipped with interpretive schemes and modularly connected with earlier confirmed theories. The symbolic universe can be defined in purely mathematical terms, typically by a set-theoretical and functional description of a set of systems and transformations upon them. The interpretive schemes are obtained by selecting a subset of the symbolic systems and focusing on a few quantitative attributes that are mutually related according to the laws of the symbolic universe. These schemes are chosen so that the associated quantities correspond to measurable quantities. They are blue prints for possible experiments in the laboratory. The procedures of measurement are dictated by theoretical modules to which the relevant quantities belong. These modules are structurally related to the theory under consideration, either because they play a role in the definition of its symbolic universe or because they are valid approximations of this theory for the purpose of measurement.

The symbolic universe without the interpretive schemes is just a mathematical theory. The interpretive schemes should be regarded as an essential part of a physical theory, even though they evolve in time when physicists learn how to better apply the theory. They are themselves mathematical objects and should not be confused with the concrete realization at which they aim. In particular, one should avoid the temptation to reify schematic quantities. Their empirical meaning is not hard-wired; it may evolve together with the class of interpretive schemes. The schematic modules, which inform measurement of the schematic quantities, are essential in importing all the knowledge, tacit or not, that we have accumulated in applying earlier successful theories.

In real life, physicists do not cleanly distinguish between symbolic systems, interpretive schemes, and real experiments; and they rarely spell out modular connections. Indeed, the intuition of the working physicist implies attaching fairly concrete ideas to abstract components of the theory. In contrast, philosophers often focus on the mathematical structure of this universe, hoping that it would give us a clue about how the theory relates to the physical world. In reality, proper understanding of the nature and functioning of physical theory requires the complex of symbols, schemes, and modules (I have argued that this structure is essential to the construction, application, comparison, and communication of theories).

This is true in particular when discussing paradoxes such as the Gibbs paradox, whose paradoxical character derives from a confusion between symbols and schemes. Let us first consider the paradox in macroscopic thermodynamics. The symbolic universe is here made of a manifold of thermodynamic states described by a number of relevant parameters and their constitutive relations (transformations are paths on this manifold); it is endowed with a work form δW and a heat form δQ; it harbors geometrical, mechanical, thermometric, and calorimetric modules for volume, pressure, temperature, and heat respectively; it also includes mechanical contraptions such as (ideal) walls and pistons; and it is subjected to the two laws of thermodynamics. For these two laws, we may take the impossibility of perpetual motion of the first and second kinds (no work can be produced in a cycle of operation on a substance without consuming an equivalent amount of heat; and there are no monothermal cyclic engines). These two laws imply that the temperature *T* can be defined so that during a reversible cycle of operations on a system, ∮δQ/T=0. For a system with the independent state variables $T,\lambda_{1},\lambda_{2}\ldots$, and for a path-connected state-space, we may use this relation to define the entropy $S(T,\lambda_{1},\lambda_{2},\ldots )$ as the integral of the form δQ/T from a reference state to the state $\left(T,\lambda_{1},\lambda_{2}\ldots \right)$. This definition presupposes the independence of the state variables and their continuous variability. In addition, it requires the existence of at least one reversible transformation from the reference state to the running state.

For the reversible mixing of two gases, we may include semipermeable walls, or absorbing chemicals, or gravity in our symbolic world. For the doubling of the mass of a given gas, however, it is not clear that we can define a reversible process comparable to the slow motion of a piston. In order to alter the mass, we need to make a hole on the vessel and to inject additional mass into it. Even though the injection can be done continuously, the opening of a communication between the vessel and a reservoir looks more like a discontinuous step. This is why we cannot determine how entropy depends on the mass of a (homogeneous) gas without further assumption. We may just leave this dependence indeterminate, or we may determine it through an additional assumption. For Gibbs, the assumption is that the entropy should depend only on the total volume of the gas and not on an eventual partition of this volume. An alternative assumption is that the mixing of two identical gases should create the same amount of entropy as the mixing of two identical gases.

We still are in the symbolic universe, of which we cannot require more than mathematical consistency. At this level, the Gibbs paradox can occur only if we follow Gibbs in requiring extensive entropies. Then the mixing entropy of two identical gases has a well-defined value at variance with the mixing entropy of two gases whose difference is arbitrarily small. At the symbolic level, this jump is not truly paradoxical because from a formal mathematical point of view there is no reason to require that the thermodynamic properties of a system should be a continuous function of its chemical properties.

In order to apply the theory, we need interpretive schemes in which the relevant quantities are meant to be actually measurable. As a first consequence of this selection, the number of independent variables in the entropy function must be fitted to the empirically known number of degrees of freedom. In the case of a gas mixture, this number will change when we will learn that a substance originally regarded as chemically homogeneous truly is not. In addition, the mixing or separation procedures that occur in interpretive schemes must be realistic. This is why Boltzmann spent much time imagining such realistic procedures in his discussion of the mixing entropy. At this more concrete level, the continuity of thermodynamic properties with respect to chemical properties might seem to be a more legitimate requirement than at the symbolic level. In reality, it is devoid of meaning because the mixing entropy, qua measurable property, appears to be relative to the temporary extent of our empirical knowledge and because some of the determinations of the symbolic world, for instance the extensivity of entropy, are irrelevant at the schematic level. The Gibbs paradox is thus completely dissolved and it turns out to have emerged from a confusion between the symbolic and schematic levels.

This kind of confusion is easy to make in the case in a phenomenological theory like the older thermodynamics. By definition, in this type of theory the difference is small between the symbolic and the schematic levels. The ideal systems and processes of the symbolic world are directly conceived as idealizations or limits of concretely realizable processes, even in the statement of the two laws of thermodynamics. In his lectures on thermodynamics [47], Max Planck insisted on the need of a more abstract approach in a mathematically consistent theory, and he also warned about naive concretizations of artifacts of the mathematical construction.

With the advent of statistical mechanics, the distance between the symbolic universe and the interpretive schemes increased dramatically. There is indeed little apparent similarity between the dynamics of a large number of molecules and the thermodynamic properties of macroscopic bodies. There is no risk here of confusing the symbolic universe with the set of interpretive schemes. But there is a risk of exaggerating the freedom in the construction of the symbolic universe, and there is a persistent risk to misconceive the relation between symbolic quantities and schematic quantities. One problem is that the kinetic-molecular picture, in itself, does not contain the concepts of exchanged heat and entropy that we need in order to apply the theory to thermodynamic systems. These concepts and quantities are introduced at the schematic level only. It is true, of course, that we can proceed axiomatically and define entropy at the symbolic level through Boltzmann’s combinatorial probability or through Boltzmann’s and Gibbs’s appropriate phase-space integrals. But it is only the analysis of quasi-thermodynamic interpretive schemes that will tell us whether this defined entropy has any operational significance.

The quasi-thermodynamic schemes refer to the long-term, equilibrium behavior of the molecular system. As we saw, Boltzmann assumes that the macroscopic properties of this system reach a stable value under given macroscopic conditions after a sufficiently long time (the thermalization time), for (almost) every choice of the initial microstate compatible with these conditions. From a holistic point of view, this implies that the long-term behavior of the system can be derived from a stationary distribution in phase space. Although Gibbs (Boltzmann too, occasionally) made such distributions (microcanonical and canonical) the basis for an axiomatic construction of the symbolic universe of statistical mechanics, it is healthy to remember that the purpose of statistical mechanics is to describe the behavior of *individual* systems under the assumption that they reach a macroscopic state of equilibrium over the long run. The distributions in phase-space essentially represent the long-term behavior of a single system.

Some schematic quantities, such as the volume, pressure, and internal energy of a gas have evident counterparts in the symbolic universe: the volume may be represented by a potential wall through which the molecules are confined to a portion of space; the pressure is the resultant of the impacts of the molecules on a wall; and the internal energy is just the total kinetic energy of the molecules plus the total potential energy of their mutual forces. The temperature is given, for instance, by the average translational kinetic energy of the molecules (in proper units), although this needs to be justified by a proof of equipartition of this energy for interacting gases or more general systems. The construction of the exchanged heat and the entropy is far less trivial. It requires the consideration of interpretive schemes in which the molecular system exchanges both heat and work with the environment. As we earlier saw, Boltzmann and Einstein did so by imagining slow variations of the potential barrier that confines the system. The resulting entropy is a well-defined function of the parameters that vary during the slow deformation. Consequently, the remarks we made in the case of macroscopic thermodynamics about the schematic realization of the mixing entropy can be transposed to the statistical-mechanical case: the empirical significance of entropy-variation formulas is confined to processes for which the interpretive schemes allow for the relevant variation. In the canonical-distribution case, which best fits the situation considered in the Gibbs paradox (constant temperature and closed vessels), the mixing entropy is empirically meaningful if concrete separation is possible, and the extensivity (in the quantum case) or non-extensivity (in the classical case) of the canonical entropy formula is devoid of empirical meaning. It is just an artifact of mathematical simplicity at the symbolic level. Following van Kampen, we could stop the analysis here and declare that the Gibbs paradox, again, derives from a naive reification of the symbolic universe. The Gibbs paradox is then solved “by replacing the Platonic idea of entropy with an operational definition,” as van Kampen puts it [1] (p. 303).

But is there truly no means to compare the entropies of different masses of the same gas in statistical mechanics? There may be hope in the fact that the macroscopic predictions of statistical mechanics do not *exactly* agree with the predictions of macroscopic thermodynamics. The comparison of the predictions of two different theories requires a shared class of interpretive schemes: we must identify systems, quantities, and processes that exist in the symbolic worlds of both theories and have clear empirical counterparts. However, the laws of the two symbolic universes imply different relations between the schematic quantities. In our case, statistical mechanics allows for fluctuations that do not exist in macroscopic thermodynamics. These fluctuations are empirically accessible, at least in principle. They may offer a new way of interconnecting the equilibrium states of systems that have different Hamiltonians. For instance, it is tempting to identify the fluctuations in which all the molecules of a gas are found in the partial volume υ of its container with the equilibrium state of this gas when it is contained in a vessel of volume υ. Statistical mechanics allows us to compute the probability of such a fluctuation, and its logarithm agrees with the volume-dependence of the entropy of the gas. We are not learning anything new in this case. But we may follow Einstein and generally assume what I called the fluctuation principle: the probability of a subclass of phases of the system that agrees with the class of phases of another system in a given macrostate determines the entropy of this state. This principle leads to non-extensive entropies in the classical case and extensive entropies in the quantum case.

If we are willing to admit the fluctuation principle, the Gibbs paradox belongs to the quantum context only, and its solution then requires the blend of continuity and discontinuity inherent in quantum mechanics. If we prefer not to invoke this principle, then the Gibbs paradox belongs equally to the classical and the quantum contexts, and can be solved in both cases by recognizing conventional aspects in the definition of entropy.
